# Results from an 18 country cross-sectional study examining experiences of nature for people with common mental health disorders

**DOI:** 10.1038/s41598-020-75825-9

**Published:** 2020-11-06

**Authors:** Michelle Tester-Jones, Mathew P. White, Lewis R. Elliott, Netta Weinstein, James Grellier, Theo Economou, Gregory N. Bratman, Anne Cleary, Mireia Gascon, Kalevi M. Korpela, Mark Nieuwenhuijsen, Aisling O’Connor, Ann Ojala, Matilda van den Bosch, Lora E. Fleming

**Affiliations:** 1 European Centre for Environment and Human Health, University of Exeter Medical School, c/o Knowledge Spa, Royal Cornwall Hospital, Truro, TR1 3HD Cornwall UK; 2 Cognitive Science Hub, Department of Psychology, Univerity of Vienna, Liebiggassse 5, 1010 Vienna, Austria; 3 School of Psychology, Cardiff University, Cardiff, UK; 4 College of Engineering, Mathematics, and Physical Sciences, University of Exeter, Exeter, UK; 5 School of Environmental and Forest Sciences, University of Washington, Washington, USA; 6 Griffith University, Brisbane, Australia; 7 Barcelona Institute for Global Health, Barcelona, Spain; 8 Faculty of Social Sciences/ Psychology, Tampere University, Tampere, Finland; 9 Environmental Protection Agency, Wexford, Ireland; 10 Natural Resources Institute Finland, Helsinki, Finland; 11 School of Population and Public Health, University of British Columbia, Vancouver, Canada

**Keywords:** Psychology, Environmental social sciences, Health care

## Abstract

Exposure to natural environments is associated with a lower risk of common mental health disorders (CMDs), such as depression and anxiety, but we know little about nature-related motivations, practices and experiences of those already experiencing CMDs. We used data from an 18-country survey to explore these issues (n = 18,838), taking self-reported doctor-prescribed medication for depression and/or anxiety as an indicator of a CMD (*n* = 2698, 14%). Intrinsic motivation for visiting nature was high for all, though slightly lower for those with CMDs. Most individuals with a CMD reported visiting nature ≥ once a week. Although perceived social pressure to visit nature was associated with higher visit likelihood, it was also associated with lower intrinsic motivation, lower visit happiness and higher visit anxiety. Individuals with CMDs seem to be using nature for self-management, but ‘green prescription’ programmes need to be sensitive, and avoid undermining intrinsic motivation and nature-based experiences.

## Introduction

There is considerable evidence that contact with (safe) natural environments such as parks and woodlands (green spaces), and rivers and lakes (blue spaces), can reduce the risk of onset of common mental health disorders (CMDs) such as depression and anxiety^1^. This may, in part, be because contact with the natural world is intrinsically motivating^2,3^, i.e. enjoyable for its own sake^4^, and can both reduce negative emotions and increase positive ones^5^. However, we know relatively little about the everyday nature-related motivations, practices and experiences of those who are already experiencing CMDs. Is nature contact only good for reducing the risk of onset, or can it also help management and recovery? ‘Green care’^6^ and ‘green prescription’^7^ initiatives suggest that it might, but evidence draws largely on small-scale studies using self-selected samples^8^. As far as we are aware, there has been no large-scale examination looking at everyday green/blue space experiences by individuals currently experiencing CMDs. The current research aimed to explore these issues.

CMDs were the leading cause of disability in 2015, with depression accounting for around 50 million person years lived with disability (YLD) and anxiety around 25 million YLDs, globally^9^. Although a range of treatments are available, individuals face challenges getting access to, and/or responding to treatment, including limited availability of face-to-face psychotherapy, potential side-effects of medication, and stigma^10^. The need for safe, complementary approaches, with a low risk of side effects is widely acknowledged^11^. Evidence suggests that contact with nature might be able to be part of a package of complementary treatments, at least for some individuals.

Analysis of longitudinal data suggests people experience less psychological distress in years when they are living in greener urban areas^12^. This is supported by cross-sectional findings revealing positive associations between greener urban areas and lower antidepressant prescription rates^13^. Experimental studies find that people with CMDs get more symptom relief from a walk in natural rather than urban settings^14,15^. ‘Green care’ initiatives, including horticultural therapy, care farming, and wilderness therapy, and ‘blue care’ initiatives such as outdoor swimming and surfing, have reported similar benefits^6,16^. Consequently, there is growing interest in ‘green prescriptions’, where health care practitioners refer CMD patients to accredited blue/green care protocols, or simply recommend spending more time in nature^17^.

These schemes remain relatively small-scale, however, due to concerns about acceptability, feasibility, and generalisability of benefits beyond self-selected individuals^7^. A key aspect of depression is a ‘motivation deficit’, i.e. a difficulty in engaging in everyday activities or trying new things^18^, and thus it may be hard to encourage individuals to start visiting nature or maintain regular contact, despite being intrinsically motivated^2^. Similarly, a feature of various anxiety disorders, and sometimes depression, is ‘experiential avoidance’ where individuals try to avoid potentially anxiety-inducing thoughts, feelings, and/or activities^19^. Since public green/blue spaces may present unpredictable environmental and/or social circumstances, people with some CMDs (e.g. agoraphobia) may wish to avoid these settings, again, despite being intrinsically motivated. Given that both motivational deficit and experiential avoidance have affected the success of programmes to promote physical activity among individuals with CMDs^20^, these experiences may also undermine the success of green prescription initiatives.

Further, according to self-determination theory (SDT), feeling pressured to engage in activities by others can undermine intrinsic motivation^21,22^. Thus, feeling pressured to visit nature by friends/family, or more formally by a ‘green prescription’ from a medical professional, may be inadvertently detrimental. In the framework of SDT, there may be a shift from visiting nature because it is intrinsically enjoyable and fun, to visiting because of an internalised desire to meet the expectations of others^21^. In turn, this can lead to increased feelings of anxiety, especially if individuals feel they are failing to live up to these expectations^23^.

The current study used data from an 18-country survey on recreational contact with green/blue spaces to explore these issues. Individuals with CMDs were identified based on self-reported medication use. Further questions asked about intrinsic motivations to spend time in nature, recreational green/blue space visit frequency, experiences of the most recent visit, and perceived social pressure to spend time in nature. The survey was part of an international project that had a particular focus on blue spaces^24^, so while recreational nature visit frequency spanned both green and blue spaces, the specific visit was concerned only with the most recent blue space visit.

We tested the following hypotheses (H):

*Intrinsic motivation* Individuals will believe time in nature to be intrinsically motivating (H1a). However, given general motivational deficits among people with CMDs, their levels of intrinsic motivation will be lower than for people without CMDs (H1b).

*Nature visit frequency* Given potential experiential avoidance, nature visit frequency will be lower for people with, versus without, CMDs (H2).

*Blue space visit wellbeing* Blue space visits will be associated with high levels of positive emotions (e.g. happiness, H3a), and low levels of negative emotions (e.g. anxiety, H3b), for people both with and without CMDs. However, given motivational deficits, happiness levels will be relatively lower (H3c), and anxiety levels relatively higher (H3d) among people with, versus without, CMDs.

*Perceived social pressure* Perceived social pressure will moderate outcomes such that the greater the perceived pressure, the lower intrinsic motivation (H4a), the less likelihood of visiting nature regularly (H4b), and the lower happiness (H4c) and greater anxiety (H4d) on recent blue space visits.

## Results

### Preliminary statistics

Table 1 presents descriptive statistics. There were *n* = 2,698 (14%) respondents who reported having at least one CMD, slightly below the 17% annual figure^25^. This is likely due to the fact we focused on medication use as a proxy for CMD, and current rather than annual rates. In terms of specific conditions the frequencies were: depression (only) *n* = 910 (4.8%); anxiety (only) *n* = 1013 (5.4%); both depression and anxiety *n* = 775 (4.1%). Supporting Hypothesis 1a, intrinsic motivation to spend time in nature, for the sample as a whole, was significantly above the mid-point of 4 (*M* = 5.80, *SD* = 1.36), *t*(18,837) = 181.60, *p* ≤ 0.001, 95% CI = [1.78, 1.82], Cohen’s *d* = 1.32. This was true of all four groups (i.e., depression, anxiety, both, neither), all *t*s > 28.23; all *p*s < 0.001; all *d*s > 1.01. In terms of visit frequency, more than half of all participants in each of the four groups reported visiting nature ≥ once a week (differences between groups, i.e. Hypothesis 2, are tested below). Supporting Hypothesis 3a, overall sample happiness during the last visit was also significantly above the mid-point of 4 (*M* = 5.81, *SD* = 1.11), *t*(14,972) = 199.36, *p* ≤ 0.001, 95% CI = [1.79, 1.83], Cohen’s *d* = 1.63. Again, this was true of all four groups, all *t*s > 33.45; all *p*s < 0.001; all *d*s > 1.20. By contrast, and supporting Hypothesis 3b, visit anxiety for the whole sample was significantly below the mid-point of 4 (*M* = 2.15, *SD* = 1.44), *t*(14,970) = − 157.49, *p* ≤ 0.001, 95% CI = [− 1.88, − 1.83], Cohen’s *d* = -1.28, and for all four groups, all *t*s > 18.48; all *p*s < 001, all *d*s > − 0.75. Finally, Perceived Social Pressure (PSP) was also, on average, low (*M* = 2.41, *SD* = 1.79), and significantly below the mid-point of 4, in general *t*(17,798) = − 118.73, *p* ≤ 0.001, 95% CI = [− 1.62, − 1.57], Cohen’s *d* = − 0.89, and for all four groups independently all *t*s > 10.70; all *ps* < 0.001, all *ds* > − 0.38. As predicted, spending time in nature appeared intrinsically motivating for everyone, including those with CMDs, and this was supported by corresponding experiences of generally high levels of happiness and low levels of anxiety on the most recent visit.

**Table 1:** Sample descriptive statistics for outcomes and moderator as a function of common mental health disorder (CMD) status.

	Totals		Intrinsic motivation		≥ weekly nature visits		Happiness last visit		Anxiety during last visit		Perceived social pressure	
	N/M	(%/SD)	M	(SD)	N	(%)	M	(SD)	M	(SD)	M	(SD)
**CMDs**^**a**^												
None	16,138	(85.7)	5.85	(1.33)	9519	58.9)	5.83	(1.08)	2.07	(1.38)	2.35	(1.76)
Depression	911	(4.8)	5.64	(1.46)	487	(53.5)	5.69	(1.26)	2.41	(1.65)	2.57	(1.88)
Anxiety	1013	(5.4)	5.45	(1.56)	621	(61.3)	5.57	(1.31)	2.70	(1.71)	2.88	(1.96)
Both	775	(4.1)	5.57	(1.55)	395	(50.9)	5.76	(1.23)	2.70	(1.65)	2.73	(2.00)

^a^Self-reported doctor-prescribed medication use. Data presented are the weighted means/SDs. Due to stratified sampling the raw and weighed data are almost identical. Missing data/‘Unsure’/‘Prefer not to answer’: CMD status = 1; Intrinsic motivation = 187; Weekly visits = 5 PSP = 1039. Of the total sample 14,973 individuals reported a recent visit (79.5%), of those Missing data/‘Unsure’/‘Prefer not to answer’: Happiness = 7; Anxiety = 8. Data for covariates presented in Supplementary Materials Table S4.

### CMDs and intrinsic motivations, nature visits and nature experiences

The left hand data columns in Table 2 present model results for intrinsic motivation, visit frequency, and happiness and anxiety on the most recent visit, with the three CMD groups compared to the reference category of no reported conditions (i.e. ‘none’). Full models including covariates are presented in Supplementary Materials Tables S5 to S8.

**Table 2:** Intrinsic motivation to visit nature, likelihood of visiting nature and experiences on the most recent nature visit as a function of common mental health disorder (CMD) status and perceived social pressure to visit nature.

Intrinsic motivation	Model 1 (without perceived social pressure)				Model 2 (with perceived social pressure)			
	*B*	95% CIs		*P*	*B*	95% CIs		*P*
		*Lower*	Upper			*Lower*	Upper	
**CMD**^**a**^								
*None (ref)*	–	–	–	–	–	–	–	–
Depression only	− 0.14**	(− 0.23,	− 0.05)	*0.002*	0.03	(− 0.12,	0.19)	*0.682*
Anxiety only	− 0.33***	(− 0.42,	− 0.25)	< *0.001*	− 0.33***	(− 0.48,	− 0.18)	< *0.001*
Both	− 0.24***	(− 0.34,	− 0.14)	< *0.001*	− 0.29**	(− 0.45,	− 0.12)	*0.001*
*Perceived social pressure (PSP)*	–	–	–	–	–0.09***	(− 0.10,	− 0.08)	< *0.001*
Depression × PSP	–	–	–	–	− 0.06*	(− 0.10,	− 0.01)	*0.022*
Anxiety × PSP	–	–	–	–	0.01	(− 0.03,	0.06)	*0.603*
Both × PSP	–	–	–	–	0.03	(− 0.02,	0.08)	*0.194*
Constant	5.59				5.72			
*n*	17,570				17,570			
*R*^2^	0.07				0.08			
*F*	31.77				34.99			

≥ Weekly visits	*OR*	95% CIs		*P*	*OR*	95% CIs		*P*
		*Lower*	Upper			*Lower*	Upper	
**CMD**^**a**^								
*None (ref)*	–	–	–	–	–	–	–	–
Depression only	1.03	(0.88,	1.20)	*0.736*	0.87	(0.67,	1.12)	*0.278*
Anxiety only	1.19*	(1.02,	1.38)	*0.026*	0.89	(0.69,	1.15)	*0.374*
Both	1.00	(0.85,	1.19)	*0.958*	0.87	(0.66,	1.15)	*0.318*
*Perceived social pressure (PSP)*	–	–	–	–	1.02	(1.00,	1.04)	*0.050*
Depression × PSP	–	–	–	–	1.07	(0.98,	1.16)	*0.134*
Anxiety × PSP	–	–	–	–	1.11*	(1.02,	1.20)	*0.012*
Both × PSP	–	–	–	–	1.05	(0.97,	1.14)	*0.238*
Constant	− 0.34				− 0.36			
*n*	17,570				17,570			
*Pseudo R*^2^ (Cox & Snell)	0.09				0.09			

Happiness last visit	*B*	95% CIs		*P*	*B*	95% CIs		*P*
		*Lower*	Upper			*Lower*	Upper	
**CMD**^**a**^								
*None (ref)*	–	–	–	–	–	–	–	–
Depression only	− 0.04	(− 0.12,	0.04)	*0.325*	0.01	(− 0.12,	0.14)	*0.901*
Anxiety only	–0.17***	(− 0.24,	− 0.10)	< *0.001*	− 0.06	(− 0.19,	0.07)	*0.342*
Both	0.10*	(0.01,	0.19)	*0.031*	0.09	(− 0.06,	0.24)	*0.234*
*Perceived social pressure (PSP)*	–	–	–	–	− 0.04***	(− 0.05,	− 0.03)	< *0.001*
Depression × PSP	–	–	–	–	− 0.01	(− 0.05,	0.03)	*0.528*
Anxiety × PSP	–	–	–	–	− 0.03	(− 0.07,	0.01)	*0.103*
Both × PSP	–	–	–	–	0.01	(− 0.03,	0.05)	*0.659*
Constant	4.60				4.65			
*n*	14,012				14,012			
*R*^2^	0.20				0.20			
*F*	85.10				78.31			

Anxiety last visit	*B*	95% CIs		*P*	*B*	95% CIs		*P*
		*Lower*	Upper			*Lower*	Upper	
**CMD**^**a**^								
*None (ref)*	–	–	–	–	–	–	–	–
Depression only	0.17**	(0.07,	0.28)	*0.001*	− 0.25**	(− 0.42,	− 0.09)	*0.003*
Anxiety only	0.20***	(0.11,	0.30)	< *0.001*	− 0.20*	(− 0.35,	− 0.04)	*0.017*
Both	0.24***	(0.12,	0.35)	< *0.001*	− 0.05	(− 0.24,	0.13)	*0.576*
*Perceived social pressure (PSP)*					0.18***	(0.17,	0.19)	< *0.001*
Depression × PSP	–	–	–	–	0.15***	(0.09,	0.20)	< *0.001*
Anxiety × PSP	–	–	–	–	0.13***	(0.08,	0.17)	<*0* *.001*
Both × PSP	–	–	–	–	0.09**	(0.04,	0.14)	*0.001*
Constant	0.98				0.86			
*n*	13,975				13,975			
*R*^2^	0.22				0.28			
*F*	71.26				89.72			

*p < 0.05, **p < 0.01, ***p < 0.001.

^a^Self-reported doctor-prescribed medication use. Different *n*s are due to missing data on predictor or outcome variables, with lower ns for the visit experiences due to only n = 14,973 people visiting a relevant location in the last four weeks. All analyses control for: sex, age, perceived financial strain, employment status, marital status, number of children in household, having a long-term limiting illness, smoking status, alcohol use, seasonal wave and country. Analyses for visit outcomes also controlled for number of companions, presence of dog, transport mode, travel time, visit duration and happiness or anxiety ‘yesterday’ (depending on outcome). Full models including all covariate data are available in Supplementary Materials.

### Intrinsic motivations

Although all groups were above the mid-point (H1a), supporting H1b, those with depression (only) (*B* = − 0.14, 95% CI = [− 0.23, − 0.05], *p* = 0.002), or anxiety (only) (*B* = − 0.33, 95% CI = [− 0.42, − 0.25], *p* < 0.001), and those with both (*B* = − 0.24, 95% CI = [− 0.34, − 0.14], *p* < 0.001), reported significantly lower levels of intrinsic motivation for spending time in nature, compared to no reported condition.

### Visiting nature at least once a week

Contrary to H2, those with anxiety (OR = 1.19, 95% CI = [1.02, 1.38], *p* = 0.026) were significantly more likely to visit nature weekly compared to no reported condition. There was no significant difference in visit likelihood for those with depression (OR = 1.03, 95% CI = [0.88, 1.19], *p* = 0.736) or both conditions (OR = 1.00, 95% CI = [0.85, 1.19], *p* < 0.001) compared to no condition.

### Happiness and anxiety on the most recent blue space visit

Contrary to H3c, compared to those with no reported conditions, those with depression reported similar levels of happiness (*B* = − 0.04, 95% CI = [− 0.12, 0.04], *p* = 0.325), and those with both depression and anxiety reported higher happiness (*B* = 0.10, 95% CI = [0.01, 0.19], *p* = 0.031). Only those with anxiety experienced, as predicted, lower happiness on the most recent visit (*B* = − 0.17, 95% CI = [− 0.24, − 0.10], *p* < 0.001). Supporting H3d, however, compared to those with no reported conditions, visit anxiety was higher for all three CMD groups: depression (*B* = 0.17, 95% CI = [0.07, 0.28], *p* = 0.001); anxiety (*B* = 0.20, 95% CI = [0.11, 0.30], *p* < 0.001); both (*B* = 0.24, 95% CI = [0.12, 0.35], *p* < 0.001).

### Potentially moderating role of perceived social pressure (PSP)

The right hand columns in Table 2 add PSP and the interactions with the three CMD groups to the first set of models. Again, full models including covariates are presented in Supplementary Materials Tables S5 to S8.

### Intrinsic motivations

Supporting H4a, PSP was associated with lower intrinsic motivation, even for those with no reported conditions. For each unit increase in PSP there was a 0.09 decrease in intrinsic motivation towards visiting natural spaces (*B* = − 0.09, 95% CI = [− 0.10, − 0.08], *p* < 0.001). The negative association was even stronger for people with depression (*B* = − 0.06 (95% CI = [− 0.10, − 0.01], *p* = 0.022), such that each unit increase in PSP was associated with an additional 0.06 decrease in intrinsic motivation, over and above the 0.09 decrease among those without conditions (i.e. total = − 0.09 + − 0.06 = − 0.15). The interactions between PSP and anxiety *(B* = 0.01 (95% CI = [− 0.03, 0.06], *p* = 0.603) and both depression/anxiety (*B* = 0.03 (95% CI = [− 0.02, 0.08], *p* = 0.194), were not significant. Each unit increase in PSP for those with anxiety was associated with a − 0.08 (i.e. − 0.09 + 0.01) decrease in intrinsic motivation, and for those with both conditions with a − 0.06 (i.e. − 0.09 + 0.03) decrease. In other words, there was a decrease but it was not larger than that already shown by those without conditions.

### Visit frequency

Contrary to H4b, PSP was associated with a marginally greater likelihood of visiting nature ≥ once a week, for those without conditions (OR = 1.02, 95% CI = [1.00, 1.04], *p* = 0.050). The interactions for those with depression (OR = 1.07, 95% CI = [0.98, 1.16], *p* = 0.134), and both conditions (OR = 1.05, 95% CI = [0.97, 1.14], *p* = 0.238) were not significant, suggesting that PSP tended to increase visit likelihood for these individuals in the same was as those without conditions. Those with anxiety, however, were significantly more likely to visit ≥ once a week than those without conditions, as PSP increased (OR = 1.11, 95% CI = [1.02, 1.20], *p* = 0.012).

### Happiness and anxiety on most recent blue space visit

Supporting H4c, even for those without conditions, each unit increase in PSP was associated with a *B* = − 0.04 (95% CI = [− 0.05, − 0.03], *p* < 0.001) decrease in visit-related happiness. That none of the interactions between PSP and CMD group were significant suggests that everyone had a similar negative association (depression: *B* = − 0.01, 95% CI = [− 0.05, − 0.03] *p* = 0.528; anxiety*: B* = − 0.03, 95% CI = [− 0.07, 0.01], *p* = 0.103; and both: *B* = − 0.01, 95% CI = [− 0.03, 0.05], *p* = 0.659).

**Figure 1:**
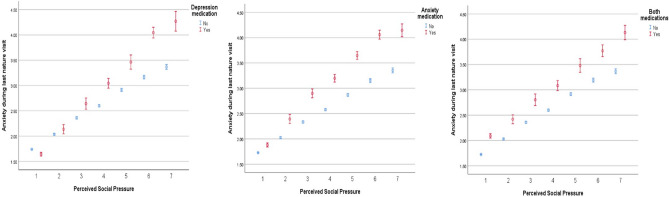
Estimated anxiety (unstandardized coefficients, 95% Confidence Intervals) during last nature visit as a function of perceived social pressure for each CMD group. Estimates are based on models controlling for: sex, age, perceived financial strain, employment status, marital status, number of children in household, having a long-term limiting illness, smoking status, alcohol use seasonal wave and country; and visit-related factors, number of companions, presence of dog, transport mode, travel time, visit duration, and anxiety ‘yesterday’.

Finally, supporting H4d, for those without conditions, each unit increase in PSP was associated with a *B* = 0.18 (95% CI = [0.17, 0.19]) increase in visit-related anxiety. This time, the interactions between PSP and CMD group were all significant (depression: *B* = 0.15 (95% CI = [0.09, 0.20], *p* ≤ 0.001; anxiety: *B* = 0.13, 95% CI = [0.08, 0.17], *p* ≤ 0.001; and both: *B* = 0.09 (95% CI = [0.04, 0.14], *p* = 0.001). These patterns are presented in Fig. 1. As PSP increases for those without conditions, anxiety also increases (blue points). However, the relationship is even steeper for all CMD groups (red points), a synergistic effect suggesting that those with CMDs who feel pressured are also susceptible to feeling greater anxiety during visits.

### Covariates

Due to space constraints results for covariates are presented and discussed in Supplementary Materials section 3.

## Discussion

To our knowledge, this is the first multi-country analysis of nature-related motives, visits, and wellbeing experiences of people suffering from common mental health disorders (CMDs) such as anxiety and depression. We found that intrinsic motivation to spend time in nature was generally high, although lower among individuals with CMDs, consistent with a general motivational deficit^18^. The majority of individuals with CMDs visited nature at least once a week, and contrary to experiential avoidance^19^, those with depression were just as likely, and those with anxiety more likely, to visit compared to those with no conditions. Consistent with research suggesting natural settings are ‘calming’, ‘stress relieving’^26^, and can help reduce negative, ruminative thoughts^26^, there was a tendency to report high levels of happiness, and low levels of anxiety, during recent blue space visits across the entire population; though experiences were slightly less positive among individuals with CMDs. Finally, consistent with self determination theory^21^ perceived social pressure (PSP) to visit green/blue spaces was associated with lower intrinsic motivation among all groups, with the association for those with depression particularly pronounced. Higher PSP was also associated with lower visit happiness, and higher visit anxiety, especially for those with CMDs. However, PSP was associated with greater likelihood of visits, especially among those with anxiety. That intrinsic motivation to spend time in nature was high for people with CMDs and more than half were visiting nature ≥ once a week, suggests that many may be using nature for affect-regulation purposes^27^. Nevertheless, those with anxiety (in particular) reported lower visit happiness than those without conditions, and all three CMD groups reported higher anxiety. Although this may reflect lower intrinsic motivation (as predicted), one issue may be our focus on blue spaces, which offer potential threats (such as drowning) which may be particularly salient for people with anxiety. Although we think this unlikely, given most visits involved walking near blue spaces, further research focusing on various natural settings for people with different types of CMD is warranted. Another possibility may be that their experiences were genuinely less positive on average due to area-based factors. CMD rates are higher in low-income neighbourhoods^9^, and deprived areas tend to have lower quality natural environments^28^. Most blue space visits are local, and visits to poorer quality areas are associated with less positive experiential wellbeing outcomes^29^. Thus, individuals with CMDs may be reporting less positive experiences, in part, because they occur in poorer quality natural spaces, potentially exacerbating socio-economic related mental health inequalities^30^.

That higher PSP was associated with a greater likelihood of visiting nature, especially for those with anxiety, but lower visit-related happiness, and greater visit-related anxiety, suggests that although some (perceived) pressure may be effective at getting people out, it may undermine intrinsic pleasure from visiting nature^31^. However, due to the cross-sectional nature of the survey, we are unable to determine causality. It may be that this instead reflects less motivated people, who experience less positive wellbeing outcomes, going out to please others. More detailed longitudinal work is needed in the ‘green prescription’ field to unpack this issue.

### Limitations

Although our sample was collected by an international polling company and was representative by age, gender and region within each country, we are not able to claim that the sample was fully representative in the respective countries. Moreover, the within country samples were also too small to test our hypotheses for each country separately. Further studies using larger samples which are able to be fully representative, such as Natural England’s, Monitor of Engagement with the Natural Environment (MENE) survey (https://www.gov.uk/government/collections/monitor-of-engagement-with-the-natural-environment-survey-purpose-and-results) are needed across multiple countries to explore the generalisability of the current findings across different geographical and cultural contexts. We also recognise that depression/anxiety are not the only CMDs, and that CMDs are on a spectrum^32^, so the current findings are only meant as a first exploration and are by no means definitive. Further, all the data were self-reported, and we were unable to validate people’s medication status or nature experiences. Although our prescription item is widely used^33^, it was also unable to account for length of use, dosage, access to other supporting services etc. or identify individuals who (a) meet criteria for a clinical diagnosis but are not currently receiving pharmaceutical treatment, (b) have particularly severe conditions, or (c) might be taking these drugs to help manage other conditions. Clearly, objective data on clinical diagnoses and treatment stage would be an important step for future research, especially as symptom severity and stage of treatment could be a critical factor in the success of green prescription uptake and adherence^34^. We are also aware that the explanatory power of our models was small (intrinsic motivation, visit likelihood) to moderate (visit experiences), and thus CMD status is only playing a very small role in these outcomes; there remain many other variables, beyond even our extensive set of covariates, which it is important to consider in improving our understanding of these outcomes. We also recognise that single items for measuring e.g. intrinsic and extrinsic motivation are not as robust as multi-item scales, but there is an inherent trade-off when collecting data from large-samples. Moreover, we recognise that we are assuming linearity in the response options for our outcome variables, when technically they could be considered ordinal scales. Nevertheless, it has long been recognised that findings are robust to this assumption for these kinds of dependent variables with results using linear and ordinal analyses producing essentially the same outcomes, with the linear approaches being far easier to interpret^35^. Finally, more in-depth qualitative work could enrich our understanding of whether and how people with CMDs are deliberately visiting nature for symptom self-management and how engagement with green/blue prescription programmes might affect their motivations and experiences^34^.

## Conclusions

Many individuals with CMDs are motivated to visit nature, and derive psychological benefits from such visits, though area level environmental inequalities may be undermining their potential for even better experiences. Nature based programmes such as ‘green prescriptions’ are becoming more prevalent. Our data suggest that perceived pressure to visit nature may increase visit frequency, but at the cost of undermining intrinsic motivation and the emotional benefits that might be achieved. Careful techniques to discuss accessing nature as a means of self- or supported-management (e.g. motivational interviewing^36^), need to be integrated into these programmes if they are to offer clients the best support.

## Method

Ethical approval for the methods, content and data management of the survey was granted by the University of Exeter’s College of Medicine and Health Research Ethics Committee (Ref: Aug16/B/099).

### Data

Data were drawn from the BlueHealth International Survey (BIS^24^). Respondents were registered in fourteen European countries (Bulgaria, Czech Republic, Estonia, Finland, France, Germany, Greece, Italy, Ireland, Netherlands, Portugal, Spain, Sweden, United Kingdom), plus Canada and three other non-European regions: Queensland (Australia), Hong Kong (China) and California (USA). The survey was administered by YouGov, an international market research company through their online panels of respondents. Further details on YouGov’s sampling approach and their adherence to international industry standards for data collection and storage can be found here https://yougov.co.uk/about/panel-methodology/, which includes a link to their detailed response to the World Association for Social, Opinion and Market Research (ESOMAR) guidelines on Online research.

The BIS was broadly concerned with people’s experiences of the natural world and a range of health and well-being outcomes. There were seven main sections including: (1) Subjective well-being; (2) Visit frequency to a wide range of different natural environments; (3) Blue spaces in the local area and childhood experiences; (4) The most recent visit to a blue space; (5) Water quality; (6) Health and well-being generally; and (7) Demographics. Full methodological details are available on the Open Science Framework website^37^. For current purposes it is important to note that while some questions (e.g. visit frequency) asked about peoples’ contact with a wide range of green (e.g. parks, woodlands, uplands) and blue (e.g. rivers, lakes, coasts) spaces, other questions, e.g. about the most recent visit, related only to blue spaces. Data will be made freely available on the internet in accordance with the European Union’s Open Data strategy after a suitable embargo period.

### Sampling

Samples of ~ 1000 respondents representative on sex, age, and geographical location were obtained from each country/region (total *n* = 18,838). To account for seasonal biases, sampling was undertaken in four waves between June 2017 and April 2018. All analyses used weights, created by YouGov, which accounted for selection, non-response, and population biases.

### Measures

Full details of all variables are presented in Supplementary Tables S2 and S3.

#### Common mental health disorders (CMDs)

Our indicator of whether someone was currently experiencing a CMD was based on the following question: “*During the past two weeks, have you used any medicines for any of the following conditions that were prescribed for you by a doctor*? *Please select all that apply*”, with ‘yes’/’no’ response options. Alongside physical health conditions, e.g. back/neck pain, were two mental health conditions: ‘*depression*’ and ‘*tension and anxiety*’. The question was taken from the European Health Interview Survey (Eurostat, 2013), and we created three CMD groups: (a) those with *depression*, i.e. taking antidepressants; (b) those with *anxiety*, i.e. those taking anxiolytics; and (c) those with both depression and anxiety. We recognise that this is only a proxy measure and, among other limitations, may miss people with CMDs not currently on medication (see “Discussion”). Medication data was missing for one participant.

#### Intrinsic motivation

Adapting items from a measure used in the physical activity domain (“I find exercise fun” and “I enjoy my exercise sessions”)^38^, a single-item asked participants the extent to which participants felt the following statement was true for them: “*I find visiting green and blue space enjoyable or fun*”, with responses on a seven-point scale from ‘not at all’ (1) to ‘very true’ (7). We suggest that similarly to exercise, visiting natural spaces may be considered under the umbrella of activities that serve to increase health and wellbeing. As such it is proposed that adapting a measure of motivation from the physical activity domain was appropriate. 187 participants responded ‘unsure’ and were classed as ‘missing’.

#### Visiting nature ≥ once a week

Participants were asked how often they had made recreational visits to a range of natural environments, including both green (vegetated) and blue (inland/coastal water) spaces, in the last four weeks (See Supplementary Table S1 for the full list) using four categorical response options (‘not at all in the last four weeks’, ‘once or twice in the last four weeks’, ‘once a week’, ‘several times a week’). The last four weeks was chosen as a recall period based on previous leisure visit surveys. ‘Not at all’ and ‘once/twice’ were collapsed together, and ‘once a week’ and ‘several times’ were collapsed together to provide a binary ‘yes’/’no’ indication of at least (≥) weekly nature visits, an important threshold in previous studies^39^. Nature visit frequency data was missing for five participants.

#### Blue space visit wellbeing

Consistent with the aims of the BlueHealth project, participants were asked to describe their most recent visit to any type of blue space within the last four weeks (see Supplementary Table S1 for taxonomy). 79.4% (n = 14,973), had made at least one such visit. Of these the majority involving walking, especially along footpaths and promenades. Other visits included sunbathing, swimming, playing with children, watersports, and ice skating (in winter months). Detailed analysis of visit type will be presented elsewhere. For current purposes the most important aspect was participant’s recalled experiential wellbeing in terms of the degree to which they agreed with the following statements: “It made me feel happy” and “It made me feel anxious”, with response options from ‘strongly disagree’ (1) to ‘strongly agree’ (7). These items were adapted from the OCED^40^ measures of positive and negative experiential wellbeing respectively. Of the 14,973, seven had missing data for happiness and eight people had missing data for anxiety.

#### Perceived social pressure

Perceived social pressure (PSP) was assessed using a single item adapted from the same motivation measure as for intrinsic motivation (“I feel under pressure from friends/family to exercise”^38^. Participants were asked the extent to which they felt the following statement was true for them: “*I sometimes feel pressured by others (e.g. partner, friends) to visit green and blue spaces*”, with responses from ‘not at all’ (1) to ‘very true’ (7). 1,038 participants responded ‘unsure’ and were classed as ‘missing’, alongside 1 person with missing data.

#### Sociodemographic and individual level controls

Regression analyses were adjusted for a number of socio-demographic indicators that have been found to be associated with nature visits and/or mental health in previous research. For full details and rationale see https://bit.ly/BIS-Technical-Report (section 4.7). These included: sex (male = reference, female); age (18–29, 30–39, 40–49, 50–59, 60 +  = reference); perceived financial strain (finding it very difficult on present income = reference, finding it difficult on present income, coping on present income, living comfortably on present income, don’t know); employment status (employed, other = reference); marital status (married or in civil partnership, not married = reference); number of children in the household (none = reference, one, two or more); having a long-term limiting illness (yes, no = reference); smoking status (current smoker, previously smoked, never smoked = reference); and alcohol use (less than monthly = reference, up to once a week, and up to daily). Seasonal wave (spring = reference, summer, autumn, winter), and country (reference = UK) were included to control for seasonal and country level effects. Raw data included ‘missing’s is presented in Supplementary Table S4.

Analyses for visit-related ‘happiness’ and ‘anxiety’ also controlled for visit-related factors that have been shown to be important for visit wellbeing in previous studies^29^ including: number of companions; presence of dog (yes, no = reference); mode of transport to get to destination (private = reference, public, walking/cycling, other); travel time to destination (in minutes; 0–14 = reference, 15–29, 30–59, 60–119, 120 +); and visit duration (in minutes; 10–20 = reference, 30–50,60–80, 90–110, 120–170, 180 +). Finally, to reduce the possibility that visit-related happiness and anxiety was merely due to general affective disposition, we also controlled for happiness and anxiety on the previous day as measures of general levels of experiential wellbeing^40^. Raw data included ‘missing’s is presented in Supplementary Table S4.

### Analyses

Analyses were conducted in IBM SPSS Statistics 25. To test H1a and H3/b, one-sample *t*-tests were conducted comparing the mean scores for the whole sample, and for sub-samples as a function of CMD group, to the scale-mid-points. Remaining hypotheses were tested using a series of linear and logistic (for the binary outcome of visit ≥ once week) regression models, with perceived social pressure (PSP) and interaction terms included to test the potential moderation effect of PSP (H4). Fully adjusted models are presented above with unadjusted and partially adjusted models presented in Supplementary Materials Table S5 to S8. Variance inflation factor (VIF) tests for all analyses suggested no evidence of multicollinearity between covariates in any of the analyses (Supplementary Materials Table S10). Country was added as a fixed effect to control for potential within-country clustering. This is similar to a random intercept model for country but with the advantage that inference on the relationship between country and outcomes is more straightforward. There were insufficient cases of medication use in each country to robustly explore country-specific medication effects (random slopes). Although we considered a matched-control design for the current analyses, i.e. only comparing those with CMDs to a reduced sample who were not on medication but similar in other respects, such designs tend to produce similar results to full sample analyses, but with reduced power^41^. Preliminary analysis suggested this was also the case here, so the full sample was retained. Loss of data due to missing/unsure/prefer not to answer responses (especially for the PSP measure) resulted in full sample (n = 18,838) models of n = 17,570 for intrinsic motivation and visits weekly (i.e. 93.3% included), and visit sub-sample (n = 14,973) analyses with n = 14,012 (93.6%) for visit happiness and n = 13,975 (93.3%) for visit anxiety. Of note, this reduced visit only sample was almost identical in terms of CMD status and demographic composition to the full sample (Supplementary Table S9), reducing the risk of bias. To aid comparability across Models 1 (without PSP) and Models 2 (with PSP), both models only used the sample with PSP data.

### The relationships between covariates and main study outcomes

Below we present a brief consideration of the significant relationships between the covariates included in the final, fully adjusted models for each outcome variable. These are presented in Tables S5 to S8 of the supplementary materials document.

### Intrinsic motivation to visit natural spaces

Intrinsic motivation (IM) was significantly higher in females compared to males (*B* = 0.19, 95% CI = [0.15, 0.23], *p* < 0.001), and lower for those participants aged 18–29 (*B* = − 0.22, 95% CI = [− 0.19, − 0.26], *p* < 0.991) and 30–39 (*B* = − 0.14, 95% CI = [− 0.21, − 0.07], *p* < 0.001) years. Perceived income was also associated with IM, such that those who felt that they were comfortable (*B* = 0.23, 95% CI = [0.13, 0.32], *p* < 0.001) or coping (*B* = 0.16, 95% CI = [0.07, 0.24], *p* < 0.001) also reported higher IM compared to those who were finding their present financial situation difficult or very difficult. This may indicate that capacity for enjoyment of nature is reduced when other more practical issues are of concern, such as financial anxiety. Those reporting that they ‘didn’t know’ how they perceived their financial situation reported lower IM (*B* = − 0.22, 95% CI = [− 0.42, − 0.02], *p* = 0.033).

Both being married/cohabiting (*B* = 0.19, 95% CI = [0.15, 0.23], *p* < 0.001), and employed (*B* = 0.05, 95% CI = [0.00, − 0.10], *p* = 0.031) were positively associated with higher IM. Enjoyment of natural spaces may be increased when visiting with a loved one, and employment status may serve here as a proxy or reflection of perceived financial situation, again potentially reflecting cognitive capacity for enjoyment in the absence of perceived financial pressure. Wave was also associated with IM; as perhaps may be expected, IM was lower in winter (*B* = − 0.10, 95% CI = [− 0.05, − 0.14], *p* < 0.001) and spring (*B* = − 0.06, 95% CI = [− 0.12, − 0.01], *p* = 0.021), compared to Summer. It is logical that enjoyment would be higher in seasons that are typically associated with milder weather. Up to daily alcohol use was also positively associated with IM. This could be a result of the social interaction that may accompany regular alcohol intake and be associated with visits for recreational purposes and therefore increased enjoyment. Finally, compared to the UK, Bulgaria (*B* = 0.53, 95% CI = [0.42, 0.64], *p* < 0.001), California (*B* = 0.13, 95% CI = [0.02, 0.24], *p* = 0.025), Estonia (*B* = 0.14, 95% CI = [0.03, 0.26], *p* = 0.015), Finland (*B* = 0.16, 95% CI = [0.05, 0.27], *p* = 0.005), Germany (*B* = 0.13, 95% CI = [0.02, 0.25], *p* = 0.020), Greece (*B* = 0.34, 95% CI = [0.23, 0.46], *p* < 0.001), Portugal (*B* = 0.45, 95% CI = [0.33, 0.56], *p* < 0.001) and Sweden (*B* = 0.12, 95% CI = [0.01, 0.23], *p* = 0.027) all reported significantly higher IM. In contrast, Canada (*B* = − 0.22, 95% CI = [− 0.33, − 0.11], *p* < 0.001), Hong Kong (*B* = − 0.53, 95% CI = [− 0.65, − 0.41], *p* < 0.001), Ireland (*B* = − 0.15, 95% CI = [− 0.26, − 0.04], *p* = 0.007) and the Netherlands (*B* = -0.25, 95% CI = [− 0.36, − 0.14], *p* < 0.001) all reported lower IM. We have no clear idea why this should be the case.

###  ≥ Weekly visits to nature

Females were significantly less likely to visit nature at least weekly compared to males (OR = 0.85, 95% CI = [0.80, 0.91], *p* < 0.001). Odds of visiting weekly were also lower for all age groups compared to those aged 60 + years (OR range between 0.76 and 0.86, all *p’s* ≤ 0.01). Perceived income was also associated with weekly visits, such that those who felt that they were comfortable (OR = 1.65, 95% CI = [1.45, 1.97], *p* < 0.001) or were coping (OR = 1.38, 95% CI = [1.20, 1.59], *p* < 0.001) had higher odds of visiting weekly compared to those who perceived their financial situation to be very difficult, possibly due to increased time, availability and capacity for leisure activities. The odds of at least weekly visits were significantly lower in winter (OR = 0.65, 95% CI = [0.59, 0.72], *p* < 0.001) and spring (OR = 0.69, 95% CI = [0.63, 0.76], *p* < 0.001), compared to summer. This is in line with the notion that visits will be more frequent when the weather is warmer and more tourist attractions are likely to be open. Both being married (OR = 1.28, 95% CI = [1.19, 1.37], *p* ≤ 0.001), and having 1 child (OR = 1.54, 95% CI = [1.38, 1.71], *p* < 0.001) or 2 or more children (OR = 1.55, 95% CI = [1.38, 1.74], *p* < 0.001) were also associated with increase odds of at least weekly visits. This may be due to the need to take family trips out and provide activities for children. Increased alcohol use (drinking at least weekly and at least daily) was also associated of increased odds of visiting nature compared to drinking less than monthly. Finally, with the exception of California, Canada and France, participants from all countries had higher odds of visiting at least weekly compared to the UK (OR range between 4.96 and 1.36, all *p*’s < 0.05).

### Visit happiness

Visit happiness during the most recent blue visit was significantly higher in females compared to males (*B* = 0.18, 95% CI = [0.15, 0.22], *p* < 0.001), and lower for those participants aged between 18 and 29 (*B* = − 0.12, 95% CI = [− 0.18, − 0.07], *p* < 0.001) and 30 to 39 (*B* = − 0.08, 95% CI = [− 0.14, − 0.03], *p* = 0.005) years. Perceived income was also associated with visit happiness, such that those who felt that they were comfortable (*B* = − 0.11, 95% CI = [− 0.19, − 0.03], *p* = 0.001) or coping (*B* = − 0.09, 95% CI = [− 0.16, − 0.01], *p* = 0.028), or didn’t know how they felt about their financial situation (*B* = − 0.62, 95% CI = [− 0.80, − 0.44], *p* < 0.001) also reported lower visit happiness compared to those who were finding their present financial situation very difficult. A possible explanation for this is that those who are finding their financial situation very difficult are experiencing the greatest benefits from blue space when they are there^42^. Both being married/cohabiting (*B* = 0.06, 95% CI = [0.02, 0.10], *p* = 0.001), and employed (*B* = 0.06, 95% CI = [0.02, 0.09], *p* = 0.006) were positively associated with increased happiness, while having 2 children or more (*B* = − 0.07, 95% CI = [− 0.12, − 0.01], *p* = 0.018) in the household was associated with decreased happiness, potentially due to the additional stressors of supervising children on a nature visit^43^. Being a current smoker (*B* = 0.05, 95% CI = [0.00, 0.09], *p* = 0.031) was associated with increased visit happiness, however there was a negative relationship between drinking alcohol up to once a week (*B* = − 0.07, 95% CI = [− 0.11, − 0.03], *p* = 0.001) and happiness on the last blue visit. There was also a negative relationship between happiness during the recent visit and respondents who completed the questionnaire during the winter (*B* = − 0.05, 95% CI = [− 0.10, − 0.01], *p* = 0.026), compared to the summer. It is logical that happiness ratings might be affected due to either more inclement weather conditions, or a reduction in tourist industry leading to fewer activities available. Compared to the UK, Bulgaria, Estonia, Finland, France, Germany, Hong Kong, Italy and the Netherlands all reported lower happiness ratings on the most recent blue space visit (B’s range between -0.10 and -0.46, all p’s < 0.05). Greece (*B* = 0.14, 95% CI = [0.05, 0.24], *p* = 0.003) and the Czech Republic (*B* = 0.10, 95% CI = [0.01, 0.20], *p* = 0.035) reported higher happiness ratings. Those walking the dog on their most recent visit reported greater happiness (*B* = 0.12, 95% CI = [0.06, 0.17], *p* < 0.001), and those who travelled by public transport (*B* = − 0.17, 95% CI = [− 0.23, − 0.11], *p* < 0.001), compared to their own private transport reported lower happiness ratings. Length of time travelling for the visit was also associated, such that those who had travelled 120 min or more (*B* = − 0.14, 95% CI = [− 0.20, − 0.08], *p* < 0.001) to get to their destination reported lower happiness ratings. Finally, duration of the visit was also associated with visit happiness such that those on visits that lasted longer than 30 min were significantly happier (all *B*’s > 0.37, all *p*’s < 0.001).

### Visit anxiety

Visit anxiety was significantly lower in females compared to males (*B* = − 0.09, 95% CI = [− 0.13, − 0.05], *p* < 0.001), and higher for those participants aged 18–29 (*B* = 0.28, 95% CI = [0.21, 0.35], *p* < 0.001) and 30–39 (*B* = 0.15, 95% CI = [0.08, 0.23], *p* < 0.001) years compared to those aged over 60. Those in the 50–59 age bracket reported significantly lower anxiety (*B* = − 0.07, 95% CI = [− 0.14, − 0.00], *p* = 0.039) compared to those aged over 60. Perceived income was also associated with anxiety, such that those reporting that they ‘didn’t know’ (*B* = 0.37, 95% CI = [0.15, 0.60], *p* = 0.001) how they perceived their financial situation had greater visit anxiety. Being married/cohabiting (*B* = − 0.06, 95% CI = [− 0.11, − 0.01], *p* < 0.001) was associated with lower anxiety, while having 1 child (*B* = 0.17, 95% CI = [0.11, 0.23], *p* < 0.001) or 2 or more children (*B* = 0.18, 95% CI = [0.11, 0.24], *p* < 0.001) was associated with increased visit anxiety. This is consistent with the data on visit happiness, and may be due to greater responsibility for the safety of the children during the visit. As expected, anxiety yesterday (*B* = 0.15, 95% CI = [0.14, 0.15], *p* < 0.001) was related to visit anxiety, and drinking alcohol up to daily (*B* = 0.07, 95% CI = [0.01, 0.13], *p* = 0.017) was also related to increased anxiety compared to drinking less than monthly. Wave was also associated with anxiety; as perhaps may be expected, anxiety was higher during winter visits (*B* = 0.06, 95% CI = [0.00, 0.12], *p* = 0.050), compared to summer. It is logical that anxiety would be higher in seasons that are typically associated with more severe weather and activities that could be perceived as more risky such as ice skating etc. Compared to the UK, participants in the majority of countries (Canada, Estonia, Finland, Germany, Greece, Hong Kong, Ireland, Italy, Netherlands, Portugal, Queensland, AU and Spain) reported significantly greater anxiety on their recent blue visit (*B*’s range from 0.19 to 0.40, all *p*’s < 0.05) while respondents from Finland (*B* = − 0.13, 95% CI = [− 0.25, − 0.02], *p* = 0.026) reported significantly lower anxiety on their last visit. Travelling on ones ‘own steam’ (*B* = − 0.11, 95% CI = [− 0.15, − 0.06], *p* < 0.001) was associated with reduced anxiety compared to using private transport, and as expected, travel times of 30 min or greater to reach the destination were also all associated with greater anxiety (all *B*’s > 0.08, all *p*’s < 0.05) compared to a travel time of less than 15 min. Finally, consistent with the happiness ratings, visit anxiety was lower for trips that lasted longer than 60 min, suggesting that longer periods of time spent in nature may be associated with increased wellbeing benefits (B’s range from − 0.10 to − 0.31, all p’s < .0.05).

## Supplementary information

Supplementary Information.
