# Supplementary material for: Results from an 18 country cross-sectional study examining experiences of nature for people with common mental health disorders
Source: Sci Rep. 2020 Nov 6;10:19408. doi: 10.1038/s41598-020-75825-9 (PMC7648621; doi:10.1038/s41598-020-75825-9)
Supplement: Supplementary file 1 — Supplementary Information. [file 41598_2020_75825_MOESM1_ESM.pdf]

Results from an 18 country cross-sectional study examining experiences of nature for people with common mental health disorders

Michelle Tester-Jones[1], Mathew P. White[1*], Lewis R. Elliott[1], Netta Weinstein[2], James Grellier[1], Theo Economou[3], Gregory N. Bratman[4], Anne Cleary[5], Mireia Gascon[6], Kalevi M. Korpela[7], Mark Nieuwenhuijsen[6], Aisling O'Connor[8], Ann Ojala[9], Matilda van den Bosch[10] & Lora E. Fleming[1]

*Corresponding author. European Centre for Environment and Human Health, University of Exeter Medical School, c/o Knowledge Spa, Royal Cornwall Hospital, Truro, Cornwall, TR1 3HD, United Kingdom. mathew.white@exeter.ac.uk

[1]European Centre for Environment and Human Health, University of Exeter Medical School, United Kingdom

[2]School of Psychology, Cardiff University, United Kingdom

[3]College of Engineering, Mathematics, and Physical Sciences, University of Exeter, United Kingdom

[4]School of Environmental and Forest Sciences, University of Washington, USA

[5]Griffith University, Australia

[6]Barcelona Institute for Global Health, Spain

[7]Faculty of Social Sciences/ Psychology, Tampere University, Finland.

[8]Environmental Protection Agency, Ireland

[9]Natural Resources Institute Finland, Finland

[10]School of Population and Public Health, University of British Columbia, Canada

Supplementary materials

1.1 Blue and green space types

Table S1: Blue and green space types presented in the BIS questionnaire

Blue/Green	Urban/Coastal/Inland	Type
Blue	Urban coastal	Esplanade or promenade
		Pier
		Harbour or marina
Blue	Rural coastal	Sandy beach
		Rocky shore
		Cliff or headland
		Lagoon
		Open sea
Blue	Urban Inland	Fountain
		Urban river or canal
		Outdoor pool or spa
Blue	Rural Inland	Lake
		Rural river or canal
		Waterfall
		Pond, stream, or small water body

		Wetland
		Ice rink
Green	Urban	Urban park or pocket park
		Large park
		Community garden or allotment
		Playground or playing field
		Cemetery or churchyard
		Botanical garden or zoo
Green	Rural	Woodland or forest
		Farmland or arable land
		Meadow or grassland
		Mountain
		Moorland or heathland
		Country park

1.2 Independent and outcome variable descriptors

Table S2: Independent and outcome variables

Variable	Wording	Response options	Source/reference
CMDs for depression CMDs for anxiety<br><br>CMDs for both	During the past two weeks, have you used any medicines for any of the following conditions that were prescribed for you by a doctor? Please select all that apply·<br><br>Depression<br><br>Tension or anxiety<br><br>None of the above<br><br>Don't know<br><br>Prefer not to answer	Categorical Checkbox item for CMDss currently taken	Adapted from the European Health Interview Survey
Intrinsic motivation	Introduced as: And how true of you are each of these statements:<br><br>"I find visiting green and blue spaces enjoyable or fun"	1= Not at all<br><br>2<br><br>3<br><br>4 = Somewhat true<br><br>5<br><br>6	Adapted items from the BREQ-2, a measure used in the physical activity domain ("I find exercise fun" and "I enjoy my exercise sessions" Markland and Tobin, 2004)

		7= Very true<br>8 Not sure	
Frequency of visits to natural spaces within the last four weeks	Introduced as: Firstly, have a look at these green/blue spaces in inland areas/coastal areas/towns or cities/rural areas below and indicate which, if any, you have visited at least once in the last four weeks in your leisure time (refer to table 1 for full list)	1 Not at all in the last four weeks<br>2 Once or twice in the last four weeks<br>3 Once a week<br>4 Several times a week	Based on Monitor of Engagement with the Natural Environment Survey and Welsh Outdoor Recreational Survey
Recalled happiness during last blue visit	Introduced as: How much do you agree with the statements below about your visit?<br>"It made me feel happy"	1 Strongly disagree<br>2 Disagree<br>3 Slightly disagree<br>4 Neither agree nor disagree<br>5 Slightly agree<br>6 Agree<br>7 Strongly agree	Adapted from OECD wellbeing measurement items

Recalled anxiety during last blue visit	"It made me feel anxious"	1 Strongly disagree<br>2 Disagree<br>3 Slightly disagree<br>4 Neither agree nor disagree<br>5 Slightly agree<br>6 Agree<br>7 Strongly agree	Adapted from OECD wellbeing measurement items
Extrinsic motivation (Whether respondent feels pressured to visit natural environments)	"I sometimes feel pressured by others (e.g. partner, friends) to visit green and blue spaces"	1= Not at all<br>2<br>3<br>4 = Somewhat true<br>5<br>6	Adapted from the BREQ-2, a measure used in the physical activity domain ("I feel under pressure from friends/family to exercise"; Markland and Tobin, 2004).

7= Very true

8 Not sure

Table S3: Sociodemographic and individual level controls

Variable	Wording	Response options	Source/reference
Gender	n/a (coded from panel registration information in most cases	1 Female<br>2 Male	n/a
Age	n/a (coded from panel registration information in most cases	1 18-29<br>2 30-39<br>3 40-49<br>4 50-59<br>5 60+	n/a

Perceived financial situation	Which of these descriptions comes closest to how you feel about your household's income nowadays?	1 Finding it very difficult on present income<br>2 Finding it difficult on present income<br>3 Coping on present income<br>4 Living comfortably on present income<br>5 Do not know	European Social Survey
Employment status	Which of these descriptions best describes your situation (in the last seven days)? Please select only one·	1 In paid work (or away temporarily) (employee, self employed, working for your family business)<br>2 Unemployed and actively looking for a job<br>3 Unemployed, wanting a job but not actively looking for a job<br>4 In education, (not paid for by employer) even if on vacation<br>5 Doing housework, looking after children, or other persons<br>6 Retired<br>7 Permanently sick or disabled	European Social Survey

		8 In community or military service<br>9 Other<br>10 Do not know	
Marital status	Which of the following best describes your marital status now?	1 Married, in a civil union, or living with your partner (cohabiting)<br>2 Single, separated/divorced/civil union dissolved or widowed/civil partner died<br>3 Neither of these<br>4 Prefer not to answer	
Children	…and how many of these [children living in your house regularly as members of your household] are children aged under 16?	1 0<br>2 1<br>3 2<br>4 3<br>5 4<br>6 5<br>7 6<br>8 7	Monitor of Engagement with the Natural Environment survey

		9 8<br>10 9<br>11 10 or more	
Happiness yesterday	Overall, how happy did you feel yesterday?	0=Not at all to 10=Completely	OECD wellbeing measurement guidelines
Anxiety yesterday	Overall, how anxious did you feel yesterday?	0=Not at all to 10=Completely	OECD wellbeing measurement guidelines
Presence of long-standing illness or disability	Are you hampered in your daily activities in any way by any longstanding illness, or disability, infirmity or mental health problem?	1 No<br>2 Yes to some extent<br>3 Yes a lot	European Social Survey
General smoking status/behaviour	Which of these best describes your smoking behaviour? This includes rolled tobacco but not pipes, cigars or electronic cigarettes	1 I have never smoked<br>2 I have only smoked a few times<br>3 I do not smoke now but I used to<br>4 I smoke but not every day<br>5 I smoke daily<br>6 Prefer not to answer	European Social Survey
Alcohol consumption in last 12 months	In the last 12 months, how often have you had a drink containing	1 Never	European Social Survey

	alcohol? This could be wine, beer, spirits, or other drinks containing alcohol	2 Less than once a month<br>3 Once a month<br>4 2-3 times a month<br>5 Once a week 6 Several times a week<br>7 Every day<br>8 Prefer not to answer	
Survey wave	n/a	1 Jun-17<br>2 Sep-17<br>3 Dec-17<br>4 Mar-18	
Number of companions	Sum total of both adults and children on visit (including respondent)	n/a	Adapted from Monitor of Engagement with the Natural Environment survey
Walking the dog	On this visit which of these activities, if any, was the main activity you did?	1 Walking the dog<br>Walking without a dog<br>3 Nordic walking (i.e. with poles)<br>4 Running<br>5 Cycling<br>6 Horse riding	Adapted from Monitor of Engagement with the Natural Environment survey with greater input from public engagement groups

	7 Golf<br>8 Adventure sport (e.g. coasteering, climbing, paragliding, offroad driving, mountain biking)<br>9 Informal games and sport (e.g. Frisbee, bat and ball, beach ball)<br>10 Fishing (including angling, crabbing)<br>11 Hunting or shooting<br>12 Conservation activity (e.g. litterpicking)<br>13 Sunbathing<br>14 Visiting an attraction<br>15 Quiet activities (e.g. reading meditating)<br>16 Playing with children<br>17 Appreciating scenery from a car<br>18 Eating or drinking<br>19 Socialising with friends<br>20 Watching wildlife	

		21 Boating (e.g. yachting, canoeing, kayaking, pedalo/paddle boat)<br>22 Commercial boat trip (e.g. organised fishing trip, marine wildlife trip)	
Mode of transport to most recent blue visit	What form of transport did you use on this journey for the majority of the distance?	1 Personal motorised transport (e·g· car, van, motorbike)<br>2 Walking (including wheelchair use and mobility scooters)<br>3 Bicycle<br>4 Ran/jogged<br>5 Bus<br>6 Train<br>7 Taxi<br>8 Hire car<br>9 Ferry or other public boat<br>10 Other (e·g· horseback)	Adapted from the Monitor of Engagement with the Natural Environment survey

Time taken to travel to most recent blue visit	Approximately how long was your total journey time from your start point to the blue space you visited?	Free numeric response box with boxes for hours and minutes separately	Adapted from the Monitor of Engagement with the Natural Environment survey
Duration of time spent in blue visit	Approximately how much time did you spend at that blue space?	1 10 minutes<br>2 20 minutes<br>3 30 minutes<br>4 40 minutes<br>5 50 minutes<br>6 1 hour<br>7 1 hour 10 minutes<br>8 1 hour 20 minutes<br>9 1 hour 30 minutes<br>10 1 hour 40 minutes<br>11 1 hour 50 minutes<br>12 2 hours<br>13 2 hours 10 minutes<br>14 2 hours 20 minutes<br>15 2 hours 30 minutes<br>16 2 hours 40 minutes	Adapted from Monitor of Engagement with the Natural Environment survey

17 2 hours 50 minutes

18 3 hours

19 3 hours 10 minutes

20 3 hours 20 minutes

21 3 hours 30 minutes

22 3 hours 40 minutes

23 3 hours 50 minutes

24 4 hours or more

2 Supplemental results

2.1 Sample descriptives

Table S4: Sample descriptives for all outcomes and moderators as a function of CMD group and covariates

	Totals		Intrinsic motivation		≥ weekly nature visits [b]		Happiness last visit		Anxiety during last visit		Perceived social pressure	
	N/M	(%/SD)	M	(SD)	N	(%)	M	(SD)	M	(SD)	M	(SD)
**CMDs[a]**												
None	16,138	(85.7)	5.85	(1.33)	9,519	(58.9)	5.83	(1.08)	2.07	(1.38)	2.35	(1.76)
Depression	911	(4.8)	5.64	(1.46)	487	(53.5)	5.69	(1.26)	2.41	(1.65)	2.57	(1.88)
Anxiety	1013	(5.4)	5.45	(1.56)	621	(61.3)	5.57	(1.31)	2.70	(1.71)	2.88	(1.96)
Both	775	(4.1)	5.57	(1.55)	395	(50.9)	5.76	(1.23)	2.70	(1.65)	2.73	(2.00)
Missing	1	(0.0)	187	(1.00)	5	(0.0)	7	(0.10)	8	(0.10)	1039	(5.50)
**Demographic Covariates**												
Sex												
Male (ref)	9193	(48.8)	5.72	(1.38)	5665	(61.6)	5.73	1.12	2.21	(1.47)	2.54	(1.83)

Female	9645	(51.2)	5.88	(1.34)	5357	(55.5)	5.89	1.10	2.08	(1.40)	2.28	(1.74)
Missing	0	(0.0)	0	(0.00)	0	(0.0)	7	(0.00)	8	(0.10)	1039	(5.50)
Age												
18-29yrs (ref)	3509	(19.0)	5.56	(1.47)	1954	(55.7)	5.58	(1.24)	2.58	(1.59)	2.71	(1.83)
30-39yrs	3412	(18.1)	5.75	(1.37)	2096	(61.4)	5.74	(1.17)	2.43	(1.59)	2.76	(1.90)
40-49yrs	3573	(19.0)	5.84	(1.35)	2100	(58.8)	5.0	(1.06)	2.11	(1.44)	2.46	(1.83)
50-59yrs	3387	(19.0)	5.92	(1.33)	1916	(56.6)	5.1	(1.03)	1.89	(1.23)	2.24	(1.73)
≥ 60 yrs	4957	(26.3)	5.92	(1.29)	2956	(59.6)	5.90	(1.01)	1.81	(1.19)	2.02	(1.59)
Missing	0	(0.0)	0	(0.00)	0	(0.0)	7	(0.00)	8	(0.1)	1039	(5.50)
Perceived financial situation												
Very difficult (ref)	1240	(6.6)	5.72	(1.57)	613	(49.4)	5.69	(1.31)	2.24	(1.46)	2.29	(1.83)
Finding it difficult	3336	(18.0)	5.72	(1.45)	1870	(56.1)	5.76	(1.15)	2.22	(1.41)	2.38	(1.77)
Coping	8762	(47.0)	5.82	(1.32)	5210	(59.5)	5.82	(1.06)	2.11	(1.35)	2.38	(1.75)

Comfortable	5232	(28.0)	5.87	(1.29)	3203	(61.2)	5.89	(1.09)	2.12	(1.57)	2.49	(1.86)
Don’t know	266	(1.4)	5.35	(1.76)	125	(46.9)	5.01	(1.43)	2.88	(1.50)	2.65	(1.76)
Missing	2	(0.0)	2	(0.00)	1	(0.0)	8	(0.10)	9	(0.10)	1041	(5.50)
Employment status												
Employed	10,392	(55.2)	5.81	(1.32)	6154	(59.2)	5.83	(1.09)	2.21	(1.50)	2.53	(1.83)
Unemployed (ref)	8442	(44.8)	5.80	(1.41)	4866	(57.6)	5.78	(1.14)	2.07	(1.36)	2.25	(1.73)
Missing	4	(0.0)	4	(0.00)	3	(0.0)	10	(0.10)	12	(0.10)	1043	(5.50)
Married/cohabiting												
Yes	11,107	(59.0)	5.90	(1.29)	6974	(63.3)	5.90	(1.04)	2.11	(1.44)	2.47	(1.82)
No (ref)	7730	(41.0)	5.66	(1.45)	4047	(36.7)	5.68	(1.20)	2.20	(1.44)	2.31	(1.74)
Missing	1	(0.0)	1	(0.00)	1	(0.0)	8	(0.10)	9	(0.1)	1040	(5.50)
# children in household												
0	13,595	(72.2)	5.81	(1.36)	7457	(67.7)	5.81	(1.10)	2.03	(1.34)	2.25	(1.71)
1	2849	(15.1)	5.78	(1.35)	1921	(67.4)	5.83	(1.10)	2.43	(1.62)	2.76	(1.92)

≥2	2394	(13.0)	5.80	(1.41)	1644	(68.7)	5.80	(1.21)	2.43	(1.64)	2.84	(1.94)
Missing	0	(0.0)	0	(0.00)	0	(0.0)	7	(0.00)	8	(0.10)	1039	(5.50)
Long-standing limiting illness												
Yes	663	(3.5)	5.65	(1.51)	316	(48.0)	5.74	(1.26)	2.14	(1.48)	2.16	(1.65)
No (ref)	18,171	(96.5)	5.81	(1.36)	10,704	(59.0)	5.81	(1.11)	2.15	(1.44)	2.42	(1.80)
Missing	4	(0.0)	4	(0.00)	3	(75.0)	10	(0.10)	12	(0.10)	1043	(5.50)
Smoker												
Never (ref)	9693	(52.0)	5.76	(1.35)	5,555	(57.3)	5.77	(1.09)	2.17	(1.40)	2.44	(1.77)
Previous	4655	(25.0)	5.89	(1.34)	2776	(60.0)	5.85	(1.12)	1.98	(1.34)	2.20	(1.68)
Current	4311	(23.1)	5.83	(1.40)	2590	(60.1)	5.88	(1.13)	2.24	(1.58)	2.54	(1.54)
Prefer not to answer	177	(0.9)	5.42	(1.34)	100	(0.9)	5.26	(1.27)	3.12	1.69	3.20	(1.95)
Missing	2	(0.0)	2	(0.00)	1	(50.0)	8	(0.10)	9	(0.10)	1040	(5.50)
Alcohol use												
< monthly (ref)	6167	(33.0)	5.75	(1.39)	3162	(51.3)	5.80	(1.11)	2.05	(1.32)	2.65	(2.18)

Up to weekly	7506	(40.0)	5.78	(1.37)	4565	(61.0)	5.77	(1.13)	2.21	(1.45)	2.75	(2.12)
Up to daily	5046	(27.0)	5.92	(1.30)	3231	(64.0)	5.89	(1.05)	2.15	(1.53)	2.72	(2.18)
Prefer not to answer	113	(0.6)	5.35	(1.83)	61	(53.9)	4.99	(1.46)	2.52	(1.52)	2.77	(1.71)
Missing	6	(0.0)	6	(0.00)	3	(50.0)	11	(0.10)	13	(0.10)	1045	(5.50)
Wave												
Summer	4,728	(25.1)	5.85	(1.35)	2924	(62.1)	5.87	(1.08)	2.11	(1.42)	2.37	(1.78)
Autumn	4,544	(24.1)	5.81	(1.37)	2839	(62.4)	5.85	(1.11)	2.12	(1.42)	2.41	(1.80)
Winter	4,716	(25.0)	5.76	(1.37)	2569	(54.5)	5.74	(1.10)	2.19	(1.46)	2.46	(1.80)
Spring	4,850	(25.7)	5.79	(1.37)	2690	(55.4)	5.77	(1.20)	2.17	(1.46)	2.38	(1.80)
Missing	0	(0.0)	0	(0.00)	0	(0.0)	7	(0.00)	8	(0.10)	1039	(5.50)
Country												
Bulgaria	1,054	(5.6)	6.34	(1.19)	821	(78.0)	5.80	(1.30)	2.08	(1.35)	2.36	(2.80)
California, US	1,078	(5.7)	5.91	(1.36)	428	(40.0)	6.02	(1.04)	1.17	(1.51)	2.34	(1.81)
Canada	1,030	(5.5)	5.59	(1.43)	510	(50.0)	5.89	(1.12)	2.50	(1.75)	2.58	(1.86)

Czech Republic	1,080	(5.7)	5.76	(1.42)	772	(71.5)	5.95	(1.06)	1.92	(1.22)	2.66	(1.85)
Estonia	961	(5.1)	5.88	(1.36)	661	(69.0)	5.56	(1.18)	2.22	(1.34)	2.61	(1.88)
Finland	1,061	(5.6)	5.92	(1.30)	692	(66.0)	5.56	(1.12)	1.65	(1.13)	1.93	(1.96)
France	1,071	(5.7)	5.48	(1.48)	458	(43.0)	5.76	(1.12)	1.84	(1.23)	2.16	(1.61)
Germany	1,025	(5.4)	5.99	(1.27)	621	(61.0)	5.55	(1.20)	2.04	(1.33)	1.90	(1.53)
Greece	970	(5.1)	6.06	(1.26)	654	(67.0)	6.10	(0.97)	2.28	(1.36)	2.43	(1.95)
Hong Kong, CN	984	(5.2)	5.11	(1.35)	472	(48.0)	5.26	(1.05)	2.62	(1.33)	3.60	(1.62)
Ireland	1,059	(5.6)	5.65	(1.41)	626	(59.0)	5.90	(1.10)	2.41	(1.59)	2.60	(1.90)
Italy	1,063	(5.7)	5.75	(1.35)	703	(66.1)	5.73	(1.06)	2.35	(1.61)	2.63	(1.90)
Netherlands	1,062	(5.6)	5.62	(1.35)	626	(59.0)	5.48	(1.15)	1.97	(1.44)	2.04	(1.59)
Portugal	946	(5.0)	6.17	(1.10)	605	(64.0)	6.10	(0.92)	1.22.3 4	(1.51)	2.93	(1.93)
Queensland, AU	1,001	(5.3)	5.66	(1.38)	440	(44.0)	6.06	(1.00)	2.23	(1.52)	2.42	(1.79)
Spain	1,057	(5.6)	5.75	(1.36)	757	(71.6)	5.96	(1.07)	2.49	(1.68)	2.64	(1.90)
Sweden	1,069	(5.7)	5.95	(1.36)	663	(62.0)	5.78	(1.14)	1.78	(1.29)	1.91	(1.49)

United Kingdom	1,267	(6.7)	5.85	(1.28)	513	(41.0)	5.93	(0.99)	1.79	(1.12)	1.94	(1.50)
**Visit specific covariates (n = 14,973 visits)**												
# of companions	4.03	(3.31)	-	-	-	-	-	-	-	-	-	-
Missing	33	(0.2)										
Walking the dog												
Yes	1,615	(10.80)	6.08	(1.18)	1289	(79.8)	5.92	(1.08)	2.12	(1.54)	2.32	(1.85)
No (ref)	13,355	(89.20)	5.91	(1.30)	8605	(64.4)	5.80	(1.11)	2.15	(1.43)	2.45	(1.81)
Missing	3	(0.0)	0	(0.00)	2	(66.6)	7	(0.00)	8	(0.10)	767	(5.10)
Mode of transport												
Can/van (ref)	7,762	(51.9)	5.95	(1.28)	4985	(62.4)	5.94	(1.06)	2.17	(1.50)	2.48	(1.84)
Public	1,410	(9.4)	5.61	(1.50)	853	(60.5)	5.48	(1.28)	2.52	(1.60)	2.77	(1.85)
Walk/cycle	5,518	(36.9)	5.97	(1.22)	3887	(70.4)	5.72	(1.10)	2.01	(1.32)	2.31	(1.74)
Other	271	(1.8)	5.78	(1.43)	163	(60.1)	5.80	(1.30)	2.18	(1.53)	2.34	(1.82)
Missing	12	(0.1)	12	0.1	14	(11.1)	18	(0.10)	19	0.1	779	(5.20)

Travel time (minutes)												
0-14 (ref)	3,742	(25.0)	6.04	(1.18)	2451	(66.0)	5.80	(1.08)	1.90	(1.23)	2.15	(1.70)
15-29	3,644	(24.3)	6.01	(1.18)	2335	(64.1)	5.85	(1.03)	1.95	(1.25)	2.51	(1.82)
30-59	2,652	(17.7)	5.90	(1.30)	1731	(65.3)	5.85	(1.02)	2.12	(1.37)	2.51	(1.82)
60-119	2,812	(18.8)	5.84	(1.36)	1911	(68.0)	5.81	(1.16)	2.45	(1.63)	2.52	(1.85)
120+	2123	(14.2)	5.70	(1.52)	1466	(69.1)	5.73	(1.33)	2.57	(1.72)	2.35	(1.83)
Missing	0	(0.0)	0	(0.00)	4	(0.0)	7	(0.00)	8	(0.10)	767	(5.10)
Visit duration (minutes)												
10- 20 (ref)	2,896	(19.3)	5.77	(1.32)	1723	(60.0)	5.28	(1.20)	2.13	(1.34)	2.24	(1.67)
30-50	2,991	(20.0)	5.83	(1.32)	1987	(66.4)	5.69	(1.09)	2.22	(1.45)	2.51	(1.82)
60-80	3,008	(20.1)	5.89	(1.29)	2014	(67.0)	5.87	(1.03)	2.19	(1.45)	2.51	(1.82)
90-110	1,173	(7.8)	6.02	(1.28)	824	(70.2)	5.97	(1.03)	2.11	(1.47)	2.52	(1.85)
120-170	2,459	(16.4)	6.03	(1.25)	1713	(69.6)	6.04	(1.05)	2.17	(1.52)	2.56	(1.90)
180+	2446	(16.3)	6.11	(1.20)	1633	(66.8)	6.20	(0.96)	2.01	(1.42)	2.35	(1.83)

Missing	0	(0.0)	0	(0.00)	2	(0.0)	7	(0.00)	7	(0.00)	767	(5.10)
Totals	18,838	(100)	-	-	11,022	(58.5)	-	-	-	-	-	-
ONS well-being yesterday												
Happiness	6.95	(2.10)	-	-	-	-	-	-	-	-	-	-
Missing	3	(0.0)										
Anxiety	4.05	(2.82)	-	-	-	-	-	-	-	-	-	-
Missing	9	(0.1)										

Note: [a] As proxied by self-reported doctor-prescribed medication use. [b] %s for ≥ weekly visit reflect % of participants in the corresponding row. Data presented are the weighted Ns, means, SDs, which is why due to rounding some numbers do not necessarily add to precisely 100. Due to stratified sampling the raw and weighed data are almost identical.

2. 2 Unadjusted, partial adjusted and fully adjusted models for main study outcomes

Table S5: Unadjusted and partial adjusted models for intrinsic motivation to visit nature as a function of CMDs use for depression/anxiety and perceived social pressure to visit nature

	Model 1 (without perceived social pressure)				Model 2 (with perceived social pressure)			
**Intrinsic motivation**		95% CIs				95% CIs		
	B	*Lower*	Upper	*P*	*B*	*Lower*	Upper	*P*
CMDs								
None (ref)	-	-	-	-	-	-	-	-
Depression only	-0.20***	(-0.29,	-0.11)	<.001	-0.01	(-0.17,	0.14)	0.88
Anxiety only	-0.40***	(-0.49,	-0.31)	<.001	-0.38***	(-0.53,	-0.22)	<.001
Both	-0.32***	(-0.42,	-0.22)	<.001	-0.43***	(-0.60,	-0.26)	<.001
Perceived social pressure (PSP)	-	-	-	-	-0.11***	(-0.12,	-0.09)	*<.001*
Depression x PSP	-	-	-	-	-0.06*	(-0.11,	-0.01)	0.011
Anxiety x PSP	-	-	-	-	0.01	(-0.03,	0.06)	0.635
Both x PSP	-	-	-	-	0.05*	(0.00,	0.10)	0.039
Constant	5.81				6.06			
N	17,571				17,571			
R^2	.01				.03			
F	41.41				68.34			

*p < 0·05, **p < 0·01,***p < 0·001

Table S5a: Adjusted models for intrinsic motivation to visit nature as a function of CMDs use for depression/anxiety and perceived social pressure to visit nature

	Model 3 (without perceived social pressure)				Model 4 (with perceived social pressure)			
**Intrinsic motivation**	95% CIs				95% CIs			
	B	*Lower*	Upper	*P*	*B*	*Lower*	Upper	*P*
CMDs								
None (ref)	-	-	-	-	-	-	-	-
Depression only	-.14**	(-.23,	-.05)	*.002*	0.03	(-0.12,	0.19)	*.682*
Anxiety only	-.33***	(-.42,	-.25)	<*.001*	-0.33***	(-0.48,	-0.18)	<*.001*
Both	-.24***	(-.34,	-.14)	<*.001*	-0.29**	(-0.45,	-0.12)	*.001*
Perceived social pressure (PSP)	-	-	-	-	-0.09***	(-0.10,	-0.08)	<*.001*
Depression x PSP	-	-	-	-	-0.06*	(-0.10,	-0.01)	*.022*
Anxiety x PSP	-	-	-	-	0.01	(-0.03,	0.06)	*.603*
Both x PSP	-	-	-	-	0.03	(-0.02,	0.08)	*.194*
Demographic Covariates								
Sex								
Female	0.21***	(0.18,	0.25)	<*.001*	0.19***	(0.15,	0.23)	<*.001*
Age								
18-29yrs	-0.27***	(-0.34,	-0.21)	<*.001*	-0.22***	(-0.28,	-0.15)	<*.001*
30-39yrs	-0.18***	(-0.25,	-0.11)	<*.001*	-0.14***	(-0.21,	-0.07)	<*.001*

40-49yrs	-0.07*	(-0.14,	-0.01)	*.033*	-0.06	(-0.13,	0.01)	*.086*
50-59yrs	0.00	(-0.07,	0.06)	*.909*	0.00	(-0.06,	0.07)	*.890*
Perceived financial situation								
Finding it difficult	0.02	(-0.07,	0.11)	*.638*	0.03	(-0.17,	0.12)	*.524*
Coping	0.15***	(0.07,	0.24)	<*.001*	0.16***	(0.07,	0.24)	<*.001*
Comfortable	0.21***	(0.12,	0.30)	<*.001*	0.22***	(0.13,	0.31)	<*.001*
Don’t know	-0.23*	(-0.43,	-0.03)	*.022*	-0.22*	(-0.41,	-0.02)	*.033*
Employment status								
Employed	0.05*	(0.01,	0.10)	*.031*	0.05*	(0.00,	0.10)	*.036*
Married/cohabiting								
Yes	0.18***	(0.13,	0.22)	<*.001*	0.19***	(0.15,	0.23)	<*.001*
# children in household								
1	-0.04	(-0.10,	0.02)	*.232*	-0.01	(-0.07,	0.05)	*.642*
≥2	-0.05	(-0.12,	0.01)	*.127*	-0.02	(-0.08,	0.05)	*.631*
Health and wellbeing Covariates								
Happy yesterday	-	-	-	-	-	-	-	-
Anxious yesterday	-	-	-	-	-	-	-	-

Presence of long standing limiting								
Yes	-0.06	(-0.17,	0.06)	*.325*	-0.06	(-0.18,	0.05)	*.270*
Smoker								
Previous	0.02	(-0.03,	0.07)	*.413*	0.02	(-0.03,	0.06)	*.546*
Current	0.01	(-0.04,	0.06)	*.776*	0.02	(-0.03,	0.07)	*.457*
Alcohol use								
Up to once a week	-0.01	(-0.05,	0.04)	*.846*	0.01	(-0.04,	0.06)	*.733*
Up to daily	0.10***	(0.04,	0.15)	<*.001*	0.11***	(0.06,	0.17)	<*.001*
Wave								
Autumn	-0.04	(-0.10,	0.01)	*.117*	-0.04	(-0.10,	0.02)	*.149*
Winter	-0.11***	(-0.16,	-0.05)	<*.001*	-0.10***	(-0.15,	-0.04)	<*.001*
Spring	-0.07*	(-0.12,	-0.01)	*.019*	-0.06*	(-0.12,	-0.01)	*.021*
Country								
Bulgaria	0.50***	(0.39,	0.61)	<*.001*	0.53***	(0.42,	0.64)	<*.001*
California, US	0.09	(-0.02,	0.21)	*.095*	0.12*	(0.02,	0.23)	*.026*
Canada	-0.27***	(-0.38,	-0.16)	<*.001*	-0.21***	(-0.33,	-0.10)	<*.001*
Czech Republic	-0.08	(-0.20,	0.03)	*.134*	-0.03	(-0.14,	0.08)	*.642*
Estonia	0.08	(-0.03,	0.20)	*.156*	0.14*	(0.03,	0.26)	*.015*
Finland	0.16**	(0.05,	0.27)	*.005*	0.16**	(0.05,	0.27)	*.006*

France	-0.35***	(-0.46,	-0.24)	<*.001*	-0.33***	(-0.44,	-0.22)	<*.001*
Germany	0.13*	(0.02,	0.25)	*.020*	0.13*	(0.02,	0.24)	*.022*
Greece	0.31***	(0.19,	0.42)	<*.001*	0.34***	(0.23,	0.46)	<*.001*
Hong Kong, CN	-0.67***	(-0.78,	-0.55)	<*.001*	-0.53***	(-0.65,	-0.41)	<*.001*
Ireland	-0.19**	(-0.30,	-0.08)	*.001*	-0.15**	(-0.26,	-0.04)	*.007*
Italy	-0.12*	(-0.23,	-0.01)	*.037*	-0.06	(-0.17,	0.05)	*.265*
Netherlands	-0.25***	(-0.36,	-0.14)	<*.001*	-0.25***	(-0.36,	-0.14)	<*.001*
Portugal	0.36***	(0.25,	0.48)	<*.001*	0.44***	(0.33,	0.56)	<*.001*
Queensland, AU	-0.12*	(-0.23,	-0.01)	*.032*	-0.09	(-0.20,	0.03)	*.129*
Spain	-0.11	(-0.22,	0.01)	*.061*	-0.06	(-0.17,	0.05)	*.274*
Sweden	0.13*	(0.02,	0.24)	*.018*	0.12*	(0.01,	0.23)	*.030*
Visit specific covariates								
# of companions	-	-	-	-	-	-	-	-
Walking the dog								
Yes	-	-	-	-	-	-	-	-
Mode of transport								
Public	-	-	-	-	-	-	-	-
Own steam	-	-	-	-	-	-	-	-
Other	-	-	-	-	-	-	-	-

Travel time (minutes)								
15-29	-	-	-	-	-	-	-	-
30-59	-	-	-	-	-	-	-	-
60-119	-	-	-	-	-	-	-	-
120+	-	-	-	-	-	-	-	-
Visit duration (minutes)								
30- 50	-	-	-	-	-	-	-	-
60- 80	-	-	-	-	-	-	-	-
90-110	-	-	-	-	-	-	-	-
120- 170	-	-	-	-	-	-	-	-
180 +	-	-	-	-	-	-	-	-
Constant	5.59				5.72			
N	17,570				17,570			
R^2	.07				.08			
F	31.77				34.99			

*p < 0·05, **p < 0·01,***p < 0·001

Table S6: Unadjusted and partial adjusted models for ≥ Weekly visits to nature as a function of CMDs use for depression/anxiety and perceived social pressure to visit nature

	Model 1 (without perceived social pressure)				Model 2 (with perceived social pressure)			
**≥ Weekly visits to nature**	95% CIs				95% CIs			
	B	*Lower*	Upper	*P*	*B*	*Lower*	Upper	*P*
CMDs								
None (ref)	-	-	-	-	-	-	-	-
Depression only	0.79**	(0.69,	0.91)	*.001*	0.64***	(0.50,	0.81)	<*.001*
Anxiety only	1.09	(0.95,	1.26)	*.226*	0.75*	(0.59,	0.96)	*.023*
Both	0.71***	(0.61,	0.83)	<*.001*	0.57***	(0.44,	0.74)	<*.001*
Perceived social pressure (PSP)	-	-	-	-	1.05***	(1.03,	1.07)	<*.001*
Depression x PSP	-	-	-	-	1.08*	(1.00,	1.17)	*.046*
Anxiety x PSP	-	-	-	-	1.14**	(1.05,	1.23)	*.001*
Both x PSP	-	-	-	-	1.08	(0.99,	1.17)	*.072*
Constant	2.04				1.84			
N	17,570				17,570			
R^2	.002				.005			

*p < 0·05, **p < 0·01,***p < 0·001

Table S6a: Fully adjusted models for ≥ Weekly visits to nature as a function of CMDs use for depression/anxiety and perceived social pressure to visit nature

	Model 3 (without perceived social pressure)				Model 4 (with perceived social pressure)			
**Intrinsic motivation**		95% CIs				95% Cis		
	B	*Lower*	Upper	*P*	*B*	*Lower*	Upper	*P*
CMDs								
None (ref)	-	-	-	-	-	-	-	-
Depression only	1.03	(0.88,	1.20)	*.736*	0.87	(0.67,	1.12)	*.278*
Anxiety only	1.19*	(1.02,	1.38)	*.026*	0.89	(0.69,	1.15)	*.374*
Both	1.00	(0.85,	1.19)	*.958*	0.87	(0.66,	1.15)	*.318*
Perceived social pressure (PSP)	-	-	-	-	1.02*	(1.00,	1.04)	*.050*
Depression x PSP	-	-	-	-	1.07	(0.98,	1.16)	*.134*
Anxiety x PSP	-	-	-	-	1.11*	(1.02,	1.20)	*.012*
Both x PSP	-	-	-	-	1.05	(0.97,	1.14)	*.238*
Demographic Covariates								
Sex								
Female	0.85***	(0.79,	0.90)	<*.001*	0.85***	(0.80,	0.91)	<*.001*
Age								
18-29yrs	0.88*	(0.79,	0.98)	*.024*				
30-39yrs	0.86*	(0.77,	0.97)	*.012*	0.86**	(0.77,	0.97)	*.009*

40-49yrs	0.76***	(0.68,	0.85)	<*.001*	0.85**	(0.75,	0.95)	*.006*
50-59yrs	0.85**	(0.77,	0.95)	*.003*	0.76***	(0.68,	0.85)	<*.001*
					0.85**	(0.76,	0.95)	*.003*
Perceived financial situation								
Finding it difficult	1.15	(0.99,	1.34)	*.067*				
Coping	1.38***	(1.20,	1.59)	<*.001*	1.15	(0.99,	1.33)	*.076*
Comfortable	1.70***	(1.46,	1.98)	<*.001*	1.38***	(1.20,	1.59)	<*.001*
Don’t know	1.11	(0.80,	1.53)	*.528*	1.69***	(1.45,	1.97)	<*.001*
					1.10	(0.79,	1.51)	*.579*
Employment status								
Employed	0.92*	(0.85,	0.99)	*.028*				
					0.91*	(0.85,	0.99)	*.024*
Married/cohabiting								
Yes	1.28***	(1.19,	1.38)	<*.001*				
					1.28***	(1.19,	1.37)	<*.001*
# children in household								
1	1.55***	(1.40,	1.73)	<*.001*				
≥2	1.58***	(1.40,	1.77)	<*.001*	1.54***	(1.38,	1.71)	<*.001*
					1.55***	(1.38,	1.74)	<*.001*
Health and wellbeing Covariates								
Happy yesterday								
Anxious yesterday								

Presence of long standing limiting								
Yes	0.89	(0.74,	1.08)	*.234*				
					0.90	(0.75,	1.08)	*.259*
Smoker								
Previous	0.93	(0.86,	1.01)	*.096*				
Current	0.88**	(0.81,	0.96)	*.005*	0.93	(0.86,	1.02)	*.106*
					0.88**	(0.81,	0.96)	*.004*
Alcohol use								
Up to once a week	1.37***	(1.27,	1.49)	<*.001*				
Up to daily	1.61***	(1.47,	1.77)	<*.001*	1.37***	(1.26,	1.48)	<*.001*
					1.60***	(1.46,	1.75)	<*.001*
Wave								
Autumn	1.04	(0.95,	1.15)	*.389*				
Winter	0.65***	(0.60,	0.72)	<*.001*	1.04	(0.94,	1.15)	*.424*
Spring	0.70***	(0.63,	0.76)	<*.001*	0.65***	(0.59,	0.72)	<*.001*
					0.698**	(0.63,	0.76)	<*.001*
Country								
Bulgaria	4.73***	(3.84,	5.82)	<*.001*				
California, US	0.96	(0.81,	1.14)	*.631*	4.68***	(3.80,	5.76)	<*.001*
Canada	1.14	(0.96,	1.36)	*.139*	0.95	(0.80,	1.13)	*.539*
Czech Republic	3.04***	(2.52,	3.67)	<*.001*	1.12	(0.94,	1.33)	*.214*
Estonia	3.08***	(2.54,	3.74)	<*.001*	2.98***	(2.47,	3.60)	<*.001*
Finland	2.84***	(2.36,	3.41)	<*.001*	3.02***	(2.49,	3.66)	<*.001*

France	1.10	(0.93,	1.31)	*.276*	2.84***	(2.37,	3.41)	<*.001*
Germany	2.16***	(1.80,	2.59)	<*.001*	1.10	(0.92,	1.31)	*.288*
Greece	5.02***	(4.06,	6.19)	<*.001*	2.15***	(1.79,	2.58)	<*.001*
Hong Kong, CN	1.74***	(1.45,	2.09)	<*.001*	4.96***	(4.02,	6.12)	<*.001*
Ireland	2.00***	(1.67,	2.39)	<*.001*	1.66***	(1.38,	2.00)	<*.001*
Italy	3.03***	(2.50,	3.66)	<*.001*	1.96***	(1.64,	2.35)	<*.001*
Netherlands	2.04***	(1.71,	2.44)	<*.001*	2.95***	(2.44,	3.57)	<*.001*
Portugal	2.68***	(2.22,	3.24)	<*.001*	2.04***	(1.70,	2.43)	<*.001*
Queensland, AU	1.38***	(1.16,	1.65)	<*.001*	2.61***	(2.16,	3.16)	<*.001*
Spain	3.68***	(3.01,	4.49)	<*.001*	1.36**	(1.14,	1.62)	*.001*
Sweden	1.98***	(1.66,	2.36)	<*.001*	3.62***	(2.97,	4.43)	<*.001*
Visit specific covariates								
# of companions	-	-	-	-	-	-	-	-
Walking the dog								
Yes	-	-	-	-	-	-	-	-
Mode of transport								
Public	-	-	-	-	-	-	-	-
Own steam	-	-	-	-	-	-	-	-
Other	-	-	-	-	-	-	-	-

Travel time (minutes)								
15-29	-	-	-	-	-	-	-	-
30-59	-	-	-	-	-	-	-	-
60-119	-	-	-	-	-	-	-	-
120+	-	-	-	-	-	-	-	-
Visit duration (minutes)								
30- 50	-	-	-	-	-	-	-	-
60- 80	-	-	-	-	-	-	-	-
90-110	-	-	-	-	-	-	-	-
120- 170	-	-	-	-	-	-	-	-
180 +	-	-	-	-	-	-	-	-
Constant	-.39				-.36			
N	17,570				17,570			
R^2	.09				.09			

*p < 0·05, **p < 0·01,***p < 0·001

Table S7: Unadjusted and partial adjusted models for happiness on the last nature visit as a function of CMDs use for depression/anxiety and perceived social pressure to visit nature

	Model 1 (without perceived social pressure)				Model 2 (with perceived social pressure)			
**Happiness**		95% CIs				95% CIs		
	B	*Lower*	Upper	*P*	*B*	*Lower*	Upper	*P*
CMDs								
None (ref)	-	-	-	-	-	-	-	-
Depression only	-0.13**	(-0.22,	-0.04)	*.004*	-0.08	(-0.23,	0.06)	*.259*
Anxiety only	-0.28***	(-0.36,	-0.20)	<*.001*	-0.24**	(-0.38,	-0.10)	*.001*
Both	-0.06	(-0.16,	0.03)	*.204*	-0.16	(-0.32,	0.01)	*.057*
Perceived social pressure (PSP)	-	-	-	-	-0.05***	(-0.07,	-0.04)	<*.001*
Depression x PSP	-	-	-	-	-0.01	(-0.06,	0.03)	*.628*
Anxiety x PSP	-	-	-	-	0.00	(-0.04,	0.04)	*.856*
Both x PSP	-	-	-	-	0.04	(-0.01,	0.09)	*.078*
Constant	5.83				5.96			
N	14,012				14,012			
R^2	.004				.01			
F	18.56				23.79			

*p < 0·05, **p < 0·01,***p < 0·001

Table S7a: Fully adjusted models for happiness on the last nature visit as a function of CMDs use for depression/anxiety and perceived social pressure to visit nature

	Model 3 (without perceived social pressure)				Model 4 (with perceived social pressure)			
**Happiness**		95% CIs				95% CIs		
	B	*Lower*	Upper	*P*	*B*	*Lower*	Upper	*P*
CMDs								
None (ref)	-	-	-	-	-	-	-	-
Depression only	-0.04	(-0.12,	0.04)	*.325*	0.01	(-0.12,	0.14)	*.901*
Anxiety only	-0.17***	(-0.24,	-0.10)	<*.001*	-0.06	(-0.19,	0.07)	*.342*
Both	0.10*	(0.01,	0.19)	*.031*	0.09	(-0.06,	0.24)	*.234*
Perceived social pressure (PSP)	-	-	-	-	-0.04***	(-0.05,	-0.03)	<*.001*
Depression x PSP	-	-	-	-	-0.01	(-0.05,	0.03)	*.528*
Anxiety x PSP	-	-	-	-	-0.03	(-0.07,	0.01)	*.103*
Both x PSP	-	-	-	-	0.01	(-0.03,	0.05)	*.659*
Demographic Covariates								
Sex								
Female	0.20***	(0.16,	0.23)	<*.001*	0.18***	(0.15,	0.22)	<*.001*
Age								
18-29yrs	-0.15***	(-0.20,	-0.09)	<*.001*	-0.12***	(-0.18,	-0.07)	<*.001*
30-39yrs	-0.10**	(-0.16,	-0.04)	*.001*	-0.08**	(-0.14,	-0.03)	*.005*

40-49yrs	0.02	(-0.04,	0.08)	*.485*	0.03	(-0.03,	0.08)	*.370*
50-59yrs	0.03	(-0.03,	0.08)	*.372*	0.03	(-0.03,	0.08)	*.315*
Perceived financial situation								
Finding it difficult	-0.07	(-0.15,	0.01)	*.069*	-0.07	(-0.15,	0.01)	*.088*
Coping	-0.09*	(-0.16,	-0.01)	*.026*	-0.09*	(-0.16,	-0.01)	*.028*
Comfortable	-0.12**	(-0.20,	-0.03)	*.006*	-0.11*	(-0.19,	-0.03)	*.010*
Don’t know	-0.62***	(-0.80,	-0.45)	<*.001*	-0.62***	(-0.80,	-0.44)	<*.001*
Employment status								
Employed	0.06**	(0.02,	0.09)	*.006*	0.06**	(0.02,	0.09)	*.006*
Married/cohabiting								
Yes	0.05**	(0.02,	0.09)	*.004*	0.06**	(0.02,	0.10)	*.001*
# children in household								
1	-0.05*	(-0.10,	0.00)	*.033*	-0.04	(-0.09,	0.01)	*.087*
≥2	-0.08**	(-0.14,	-0.03)	*.003*	-0.07*	(-0.12,	-0.01)	*.018*
Health and wellbeing Covariates								
Happy yesterday	0.13***	0.13	0.14	<*.001*	0.13***	(0.13,	0.14)	<*.001*
Anxious yesterday	-	-	-	-				

Presence of long standing limiting								
Yes	0.06	-0.04	0.17	*.254*	0.06	(-0.05,	0.16)	*.268*
Smoker								
Previous	-0.01	(-0.05,	0.03)	.578	-0.01	(-0.06,	0.03)	*.507*
Current	0.04	(0.00,	0.08)	.065	0.05*	(0.00,	0.09)	*.031*
Alcohol use								
Up to once a week	-0.07**	(-0.11,	-0.03)	*.001*	-0.07***	(-0.11,	-0.03)	*.001*
Up to daily	-0.03	(-0.08,	0.01)	*.144*	-0.03	(-0.07,	0.02)	*.254*
Wave								
Autumn	-0.03	(-0.08,	0.01)	*.140*	-0.03	(-0.08,	0.01)	*.154*
Winter	-0.06*	(-0.10,	-0.01)	*.018*	-0.05*	(-0.10,	-0.01)	*.026*
Spring	-0.04	(-0.09,	0.00)	*.069*	-0.04	(-0.09,	0.00)	*.065*
Country								
Bulgaria	-0.12*	(-0.21,	-0.02)	*.017*	-0.10*	(-0.20,	-0.01)	*.034*
California, US	0.02	(-0.08,	0.12)	*.713*	0.03	(-0.07,	0.13)	*.502*
Canada	-0.06	(-0.16,	0.04)	*.237*	-0.03	(-0.13,	0.07)	*.525*
Czech Republic	0.07	(-0.02,	0.17)	*.133*	0.10*	(0.01,	0.20)	*.035*
Estonia	-0.25***	(-0.35,	-0.15)	<*.001*	-0.22***	(-0.32,	-0.12)	<*.001*
Finland	-0.21***	(-0.31,	-0.12)	<.001	-0.21***	(-0.30,	-0.11)	<*.001*

France	-0.18***	(-0.28,	-0.08)	<.001	-0.18***	(-0.27,	-0.08)	*<.001*
Germany	-0.38***	(-0.48,	-0.28)	<.001	-0.38***	(-0.48,	-0.28)	*<.001*
Greece	0.13*	(0.03,	0.22)	*.001*	0.14**	(0.05,	0.24)	*.003*
Hong Kong, CN	-0.42***	(-0.52,	-0.32)	*<.001*	-0.35***	(-0.46,	-0.25)	*<.001*
Ireland	0.00	(-0.09,	0.10)	*.927*	0.03	(-0.07,	0.12)	*.602*
Italy	-0.25***	(-0.34,	-0.16)	*<.001*	-0.22***	(-0.32,	-0.13)	*<.001*
Netherlands	-0.47***	(-0.57,	-0.37)	*<.001*	-0.46***	(-0.56,	-0.37)	*<.001*
Portugal	0.07	(-0.03,	0.17)	*.146*	0.09	(-0.01,	0.19)	*.064*
Queensland, AU	0.07	(-0.03,	0.17)	*.150*	0.11*	(0.02,	0.21)	*.022*
Spain	-0.08	(-0.17,	0.01)	*.089*	-0.06	(-0.15,	0.04)	*.226*
Sweden	-0.01	(-0.11,	0.08)	*.802*	-0.01	(-0.11,	0.08)	*.784*
Visit specific covariates								
# of companions	0.00	(-0.01,	0.00)	*.380*	0.00	(-0.01,	0.00)	*.375*
Walking the dog								
Yes	0.12***	(0.06,	0.17)	*<.001*	0.12***	(0.06,	0.17)	*<.001*
Mode of transport								
Public	-0.17***	-0.23	-0.11	*<.001*	-0.17***	(-0.23,	-0.11)	*<.001*
Own steam	0.03	-0.01	0.07	*.179*	0.02	(-0.02,	0.06)	*.256*
Other	-0.01	-0.14	0.12	*.885*	-0.02	(-0.15,	0.11)	*.770*

Travel time (minutes)								
15-29	0.02	(-0.03,	0.07)	*.386*	0.02	(-0.03,	0.07)	*.378*
30-59	0.02	(-0.04,	0.07)	*.561*	0.02	(-0.03,	0.07)	*.441*
60-119	-0.04	(-0.10,	0.01)	*.102*	-0.03	(-0.08,	0.03)	*.313*
120+	-0.16***	(-0.22,	-0.10)	<*.001*	-0.14***	(-0.20,	-0.08)	<*.001*
Visit duration (minutes)								
30- 50	0.36	(0.30,	0.41)	<*.001*	0.37***	(0.31,	0.42)	<*.001*
60- 80	0.54	(0.48,	0.59)	<*.001*	0.54***	(0.49,	0.59)	<*.001*
90-110	0.63	(0.56,	0.70)	<*.001*	0.63***	(0.56,	0.70)	<*.001*
120- 170	0.70	(0.64,	0.76)	<*.001*	0.70***	(0.65,	0.76)	<*.001*
180 +	0.85	(0.79,	0.91)	<*.001*	0.85***	(0.79,	0.91)	<*.001*
Constant	4.60				4.65			
N	14,012				14,012			
R^2	.20				.20			
F	85.10				78.31			

*p < 0·05, **p < 0·01,***p < 0·001

Table S8: Unadjusted and partial adjusted models for anxiety on the last nature visit as a function of CMDs use for depression/anxiety and perceived social pressure to visit nature

	Model 1 (without perceived social pressure)				Model 2 (with perceived social pressure)			
**Anxiety**		95% CIs				95% CIs		
	B	*Lower*	Upper	*P*	*B*	*Lower*	Upper	*P*
CMDs								
None (ref)	-	-	-	-	-	-	-	-
Depression only	0.32***	(0.20,	0.43)	<*.001*	-0.28**	(-0.46,	-0.10)	*.002*
Anxiety only	0.65***	(0.54,	0.75)	<*.001*	0.05	(-0.13,	0.22)	*.608*
Both	0.61***	(0.49,	0.74)	<*.001*	0.29**	(0.09,	0.48)	*.005*
Perceived social pressure (PSP)	-	-	-	-	0.26***	(0.25,	0.28)	<*.001*
Depression x PSP	-	-	-	-	0.20***	(0.15,	0.25)	<*.001*
Anxiety x PSP	-	-	-	-	0.15***	(0.11,	0.20)	<*.001*
Both x PSP	-	-	-	-	0.08**	(0.02,	0.14)	*.009*
Constant	2.09				1.47			
N	13,975				13,975			
R^2	.02				.15			
F	83.93				357.66			

*p < 0·05, **p < 0·01,***p < 0·001

Table S8a: Fully adjusted models for anxiety on the last nature visit as a function of CMDs use for depression/anxiety and perceived social pressure to visit nature

	Model 3 (without perceived social pressure)				Model 4 (with perceived social pressure)			
**Anxiety**		95% CIs				95% CIs		
	B	*Lower*	Upper	*P*	*B*	*Lower*	Upper	*P*
CMDs								
None (ref)	-	-	-	-	-	-	-	-
Depression only	0.17**	(0.07,	0.28)	*.001*	-0.25**	(-0.42,	-0.09)	*.003*
Anxiety only	0.20***	(0.11,	0.30)	<*.001*	-0.20*	(-0.35,	-0.04)	*.017*
Both	0.24***	(0.12,	0.35)	<*.001*	-0.05	(-0.24,	0.13)	*.576*
Perceived social pressure (PSP)	-	-	-	-	0.18***	(0.17,	0.19)	<*.001*
Depression x PSP	-	-	-	-	0.15***	(0.09,	0.20)	<*.001*
Anxiety x PSP	-	-	-	-	0.13***	(0.08,	0.17)	<*.001*
Both x PSP	-	-	-	-	0.09**	(0.04,	0.14)	*.001*
Demographic Covariates								
Sex								
Female	-0.14***	(-0.19,	-0.10)	<*.001*	-0.09***	(-0.13,	-0.05)	<*.001*
Age								
18-29yrs	0.36***	(0.29,	0.43)	<*.001*	0.28***	(0.21,	0.35)	<*.001*

30-39yrs	0.22***	(0.14,	0.29)	<*.001*	0.15***	(0.08,	0.23)	<*.001*
40-49yrs	-0.02	(-0.09,	0.06)	*.639*	-0.03	(-0.10,	0.05)	*.476*
50-59yrs	-0.07	(-0.14,	0.00)	*.063*	-0.07*	(-0.14,	0.00)	*.039*
Perceived financial situation								
Finding it difficult	0.06	(-0.04,	0.17)	*.225*	0.03	(-0.07,	0.13)	*.521*
Coping	0.03	(-0.07,	0.12)	*.615*	0.00	(-0.10,	0.09)	*.943*
Comfortable	0.10	(-0.01,	0.20)	*.062*	0.03	(-0.07,	0.13)	*.549*
Don't know	0.42***	(0.19,	0.65)	<*.001*	0.37**	(0.15,	0.60)	*.001*
Employment status								
Employed	0.02	(-0.03,	0.07)	*.435*	0.02	(-0.03,	0.07)	*.471*
Married/cohabiting								
Yes	-0.03	(-0.08,	0.02)	*.191*	-0.06*	(-0.11,	-0.01)	*.013*
# children in household								
1	0.22***	(0.16,	0.28)	<*.001*	0.17***	(0.11,	0.23)	<*.001*
≥2	0.25***	(0.18,	0.32)	<*.001*	0.18***	(0.11,	0.24)	<*.001*
Health and wellbeing Covariates								
Happy yesterday								
Anxious yesterday	0.17***	(0.16,	0.18)	<*.001*	0.15***	(0.14,	0.15)	<*.001*

Presence of long standing limiting								
Yes	0.02	(-0.11,	0.16)	*.747*	0.05	(-0.09,	0.18)	*.488*
Smoker								
Previous	-0.01	(-0.06,	0.05)	*.835*	0.00	(-0.05,	0.05)	*.944*
Current	0.04	(-0.02,	0.09)	*.220*	0.00	(-0.05,	0.06)	*.917*
Alcohol use								
Up to once a week	0.07**	(0.02,	0.12)	*.007*	0.05	(0.00,	0.10)	*.053*
Up to daily	0.10**	(0.04,	0.16)	*.001*	0.07*	(0.01,	0.13)	*.017*
Wave								
Autumn	0.02	(-0.04,	0.08)	*.571*	0.01	(-0.04,	0.07)	*.655*
Winter	0.07*	(0.01,	0.13)	*.017*	0.06*	(0.00,	0.12)	*.050*
Spring	0.04	(-0.02,	0.10)	*.230*	0.04	(-0.02,	0.10)	*.194*
Country								
Bulgaria	0.18**	(0.05,	0.30)	*.006*	0.12	(0.00,	0.24)	*.053*
California, US	0.16*	(0.04,	0.29)	*.012*	0.11	(-0.01,	0.24)	*.075*
Canada	0.41***	(0.28,	0.54)	<*.001*	0.31***	(0.19,	0.44)	<*.001*
Czech Republic	0.13*	(0.01,	0.26)	*.034*	-0.01	(-0.13,	0.11)	*.841*
Estonia	0.31**	(0.19,	0.44)	*.001*	0.18**	(0.06,	0.31)	*.003*

Finland	-0.11	(-0.24,	0.01)	*.068*	-0.13*	(-0.25,	-0.02)	*.026*
France	-0.04	(-0.16,	0.09)	*.581*	-0.04	(-0.16,	0.08)	*.484*
Germany	0.25***	(0.12,	0.37)	<*.001*	0.24***	(0.12,	0.37)	<*.001*
Greece	0.12	(-0.01,	0.24)	*.067*	0.08	(-0.05,	0.20)	*.220*
Hong Kong, CN	0.43***	(0.30,	0.56)	<*.001*	0.15*	(0.03,	0.28)	*.019*
Ireland	0.33***	(0.21,	0.45)	<*.001*	0.25***	(0.13,	0.36)	<*.001*
Italy	0.31***	(0.19,	0.43)	<*.001*	0.21***	(0.09,	0.33)	<*.001*
Netherlands	0.26***	(0.13,	0.38)	<*.001*	0.22***	(0.10,	0.34)	<*.001*
Portugal	0.28***	(0.16,	0.41)	<*.001*	0.15*	(0.03,	0.27)	*.016*
Queensland, AU	0.30***	(0.17,	0.42)	<*.001*	0.19**	(0.07,	0.31)	*.002*
Spain	0.49***	(0.37,	0.61)	<*.001*	0.40***	(0.29,	0.52)	<*.001*
Sweden	-0.01	(-0.13,	0.11)	*.887*	0.00	(-0.12,	0.12)	*.960*
Visit specific covariates								
# of companions	0.01*	(0.00,	0.02)	*.016*	0.01*	(0.00,	0.02)	*.015*
Walking the dog								
Yes	0.03	(-0.04,	0.10)	*.379*	0.04	(-0.03,	0.10)	*.288*
Mode of transport								
Public	0.07	(-0.01,	0.15)	*.080*	0.07	(-0.01,	0.15)	*.074*
Own steam	-0.13***	(-0.18,	-0.08)	<*.001*	-0.11***	(-0.15,	-0.06)	<*.001*

Other	-0.04	(-0.21,	0.12)	*.622*	0.00	(-0.16,	0.16)	*.972*
Travel time (minutes)								
15-29	0.00	(-0.07,	0.06)	*.887*	-0.01	(-0.06,	0.05)	*.876*
30-59	0.10**	(0.03,	0.16)	*.005*	0.08*	(0.01,	0.14)	*.018*
60-119	0.33***	(0.27,	0.40)	<*.001*	0.27***	(0.21,	0.34)	<*.001*
120+	0.42***	(0.34,	0.50)	<*.001*	0.33***	(0.25,	0.40)	<*.001*
Visit duration (minutes)								
30- 50	0.00	(-0.07,	0.07)	*.937*	-0.03	(-0.10,	0.04)	*.361*
60- 80	-0.08*	(-0.15,	-0.01)	*.031*	-0.10**	(-0.17,	-0.03)	*.004*
90-110	-0.22***	(-0.31,	-0.12)	<*.001*	-0.24***	(-0.33,	-0.16)	<*.001*
120- 170	-0.15***	(-0.23,	-0.08)	<*.001*	-0.18***	(-0.25,	-0.11)	<*.001*
180 +	-0.33***	(-0.41,	-0.26)	<*.001*	-0.31***	(-0.39,	-0.24)	<*.001*
Constant	0.98				0.86			
N	13,975				13,975			
R^2	.22				.28			
F	71.26				89.72			

*p < 0·05, **p < 0·01,***p < 0·001

Table S9: Key demographics for the full (18,838) vs. reduced (14,973) sample

	Full sample (n= 18,838)		Reduced (most recent visit only sample, n = 14,973)	
	N/M	(%/SD)	N/M	(%SD)
**CMDs**				
None	16,138	(85.7)	12911	(86.2)
Depression	910	(4.8)	684	(4.6)
Anxiety	1013	(5.4)	830	(5.5)
Both	775	(4.1)	547	(3.7)
**Demographic Covariates**				
Sex				
Male (ref)	9193	(48.8)	7477	(49.9)
Female	9645	(51.2)	7497	(50.1)
Age				
18-29yrs (ref)	3509	(19.0)	2941	(19.6)

30-39yrs	3412	(18.1)	2777	(18.5)
40-49yrs	3573	(19.0)	2761	(18.4)
50-59yrs	3387	(19.0)	2614	(17.5)
≥ 60 yrs	4957	(26.3)	3880	(25.9)
Perceived financial situation				
Very difficult (ref)	1240	(6.6)	868	(5.8)
Finding it difficult	3336	(18.0)	2572	(17.2)
Coping	8762	(47.0)	7022	(46.9)
Comfortable	5232	(28.0)	4327	(28.9)
Don’t know	266	(1.4)	182	(1.2)
Employment status				
Employed	10,392	(55.2)	8413	(56.2)
Unemployed (ref)	8442	(44.8)	6556	(43.8)
Married/cohabiting				
Yes	11,107	(59.0)	9064	(60.5)

No (ref)	7730	(41.0)	5908	(39.5)
# children in household				
0	13,595	(72.2)	10613	(70.9)
1	2849	(15.1)	2367	(15.8)
≥2	2394	(13.0)	1994	(13.3)
Long-standing limiting illness				
Yes	663	(3.5)	430	(2.9)
No (ref)	18,171	(96.5)		
Smoker				
Never (ref)	9693	(52.0)	7742	(51.7)
Previous	4655	(25.0)	3688	(24.6)
Current	4311	(23.1)	3399	(22.7)
Prefer not to answer	177	(0.9)	144	(1.0)
Alcohol use				
< monthly (ref)	6167	(33.0)	4460	(29.8)

Up to weekly	7506	(40.0)	6199	(41.4)
Up to daily	5046	(27.0)	4223	(28.2)
Prefer not to answer	113	(0.6)	86	(0.6)

Table S10: Variance inflation factors for each of the fully adjusted models

	Intrinsic motivation	≥ weekly visits	Happiness last visit	Anxiety last visit
CMDs				
Depression only	2.93	2.93	2.95	2.94
Anxiety only	3.20	3.20	3.30	3.30
Both	2.97	2.97	3.01	3.01
Perceived social pressure (PSP)	1.31	1.31	1.32	1.40
Depression x PSP	2.97	2.97	3.41	3.01
Anxiety x PSP	3.31	3.31	3.00	3.40
Both x PSP	2.97	2.97	2.99	2.98

Demographic Covariates				
Sex				
Female	1.05	1.05	1.05	1.05
Age				
18-29yrs	1.73	1.73	1.79	1.81
30-39yrs	1.87	1.87	1.92	1.93
40-49yrs	1.87	1.87	1.90	1.90
50-59yrs	1.59	1.59	1.60	1.60
Perceived financial situation				
Finding it difficult	3.20	3.20	3.47	3.43
Coping	4.81	4.82	5.55	5.40
Comfortable	4.45	4.45	5.27	5.01
Don’t know	1.20	1.20	1.21	1.21
Employment status				

Employed	1.40	1.40	1.40	1.40
Married/cohabiting				
Yes	1.24	1.24	1.26	1.25
# children in household				
1	1.27	1.20	1.21	1.21
≥2	1.24	1.27	1.30	1.30
Health and wellbeing Covariates				
Happy yesterday	-	-	1.20	-
Anxious yesterday	-	-	-	1.23
Presence of long standing limiting illness				
Yes	1.53	1.15	1.14	1.14
Smoker				
Previous	1.23	1.23	1.23	1.23

Current	1.22	1.22	1.23	1.23
Alcohol use				
Up to once a week	1.43	1.43	1.49	1.50
Up to daily	1.52	1.52	1.60	1.60
Wave				
Autumn	1.50	1.50	1.50	1.50
Winter	1.51	1.51	1.50	1.50
Spring	1.51	1.51	1.50	1.50
Country				
Bulgaria	1.76	1.75	1.95	1.50
California, US	1.73	1.73	1.72	1.72
Canada	1.72	1.72	1.68	1.70
Czech Republic	1.75	1.75	1.84	1.84
Estonia	1.68	1.68	1.80	1.80
Finland	1.75	1.75	1.90	1.90
France	1.74	1.74	1.75	1.80

Germany	1.68	1.68	1.71	1.71
Greece	1.76	1.76	2.00	2.01
Hong Kong, CN	1.78	1.78	1.94	1.93
Ireland	1.73	1.73	1.82	1.82
Italy	1.73	1.73	1.87	1.90
Netherlands	1.73	1.73	1.75	1.75
Portugal	1.70	1.70	1.87	1.90
Queensland, AU	1.71	1.71	1.80	1.79
Spain	1.74	1.74	1.93	1.83
Sweden	1.75	1.75	1.81	1.81
**Visit specific covariates**				
# of companions	-	-	1.31	1.13
Walking the dog				
Yes	-	-	1.05	1.05
Mode of transport				
Public	-	-	1.22	1.21
Walk/cycle	-	-	1.32	1.33
Other	-	-	1.05	1.05

Travel time (minutes)				
15-29	-	-	1.60	1.60
30-59	-	-	1.52	1.52
60-119	-	-	1.59	1.60
120+	-	-	1.58	1.58
Visit duration (minutes)				
30-50	-	-	1.67	1.67
60-80	-	-	1.72	1.72
90-110	-	-	1.37	1.37
120-170	-	-	1.80	1.76
180+	-	-	1.90	1.90
